# HLA allele and haplotype frequencies of eight Indian populations based on 130,518 registered stem cell donors

**DOI:** 10.3389/fimmu.2025.1528177

**Published:** 2025-02-25

**Authors:** Ute V. Solloch, Chinma Kaniyath Madhusoodhanan, Vinzenz Lange, Jürgen Sauter, Latha Jagannathan, Patrick Paul, Marcelo A. Fernández-Viña, Alexander H. Schmidt

**Affiliations:** ^1^ DKMS Group, Tübingen, Germany; ^2^ DKMS Foundation, Bangalore, India; ^3^ DKMS Life Science Lab, Dresden, Germany; ^4^ Bangalore Medical Services Trust, Bangalore, India; ^5^ Department of Pathology, Stanford University, Stanford, CA, United States

**Keywords:** HSCT, HLA, haplotype frequency, donor registry, India

## Abstract

**Introduction:**

In hematopoietic stem cell transplantation, optimal results are achieved when donors and patients are matched regarding their human leukocyte antigen (HLA) genes. Population-specific HLA allele and haplotype frequency distributions determine the probabilities to find matched donors in a stem cell donor registry of given size and ethnic composition.

**Methods:**

To evaluate the needs of Indian patients with regard to future donor recruitment, we analyzed a large data set of *n*=130,518 potential stem cell donors registered with DKMS-BMST, a Bangalore-based donor registry with nationwide donor recruitment activities. We defined 8 subpopulations by native language and state of origin of both parents. The subpopulations comprised four samples belonging to the Dravidian language family (native language: Kannada/state of origin: Karnataka, Tamil/Tamil Nadu, Telugu/Andhra Pradesh and Malayalam/Kerala), and four samples belonging to the Indo-Aryan language group (Bengali/West Bengal, Gujarati/Gujarat, Hindi/Uttar Pradesh, Marathi/Maharashtra). The precise definition of subpopulations and large sample sizes between *n*=5,808 (Telugu/Andhra Pradesh) and *n*=14,866 (Malayalam/Kerala) are strengths of our study. Our results regarding HLA allele and haplotype frequencies refine published data.

**Results and Discussion:**

In terms of genetic relatedness, we observed a division of the subpopulations into a Southern and a Northwestern Indian cluster and the Bengali/West Bengal sample which differed significantly from the seven other subpopulations. Patients from Southern Indian populations are the main beneficiaries from the DKMS-BMST registry in the current ethnic composition. A more even nationwide coverage will be achieved in the future with the opening of local recruitment offices in different parts of India.

## 1 Introduction

For many patients with severe hematological disorders such as blood cancer, a hematopoietic stem cell transplantation (HSCT) from an allogeneic donor is the only chance of a cure. In cases where a suited matched related donor is not available, an unrelated donor is searched in worldwide registries. The optimal transplantation outcomes in adult donor unrelated HSCT are achieved in patients with a donor that matches at all alleles of the human leukocyte antigen (HLA) loci HLA-A, -B, -C, and -DRB1 (8/8 match); additional matching of the HLA-DQB1 and -DPB1 loci (10/10 or 12/12 match, respectively) may further improve outcomes (1–5). Recent publications have also examined the extent to which a graft-versus-host disease (GVHD) prophylaxis based on cyclophosphamide (PTCy) may reduce or even balance the impact of one or more mismatches on the outcome of unrelated HSCT (5–8).

Because of their close linkage, the genes of the HLA complex on the short arm of chromosome 6 are inherited as haplotype blocks. HLA genes are highly polymorphic, with both allele variation and haplotype composition being population-specific. As of June 2024, the IPD-IMGT/HLA database contained 38,975 distinct HLA alleles (9). The probability of finding a matched HSCT donor depends on the HLA diversity of the patient’s ancestry population and on the availability of donors from the same or a genetically related population (10–12).

By the end of June 2024, the World Marrow Donor Association (WMDA) included more than 42.3 million potential stem cell donors and cryopreserved cord blood units in their database (13). With approximately 12.5 million donors across seven countries, DKMS is a major donor registry. More than 118,400 DKMS donors have donated hematopoietic stem cells from peripheral blood or bone marrow to patients in around 60 countries. DKMS BMST Foundation India (referred to as “DKMS-BMST”) has registered over 133,700 stem cell donors, with more than 130 having donated hematopoietic stem cells (as of the end of June 2024). Since February 1, 2025, operations of “DKMS BMST Foundation India” transitioned to “DKMS Foundation India”.

India, with a population exceeding 1.43 billion living in 28 states and eight union territories, is characterized by its immense ethnic, linguistic, and cultural diversity (14, 15). The country’s complex demographic landscape results from a long history of migratory movements and cultural exchange, particularly in the northern regions. This historical context has shaped the ethnic and linguistic composition of India (16–18). The majority of Indians speak Dravidian or Indo-Aryan languages. The Dravidian language family is mainly represented in the South Indian peninsula, which is separated from the northern parts of India by the mountains of the Vindhya range and characterized by long oceanic coastlines and was thus geographically and historically more isolated. Indo-Aryan languages, a subgroup of the Indo-Iranian language branch of the Indo-European language family, are predominant in the northern parts of India (15, 17–19). The most prominent Indo-Aryan languages today are Hindi, Bengali and Punjabi, while the Dravidian languages with the highest numbers of speakers today are Telugu, Tamil, Kannada and Malayalam.

Systematic analyses of HLA characteristics in different Indian populations are scarce. Most of the data published or documented in the Allele Frequency Net Database (AFND) are restricted to groups with very small sample sizes, a limited number of HLA loci, low-resolution HLA typing, or are based on geographically broadly defined samples (20). The largest study with published HLA data to date involved 18,220 Indian individuals, divided into 14 populations (*n*≥200) according to Indian state affiliation (21). Further studies focused on different language groups of South Indian individuals (22–27). In addition, high-resolution HLA-A, -B, -C, -DRB1 and -DQB1 allele and haplotype frequencies of privately banked Indian umbilical cord blood units were documented in the AFND for six geographically broad regions of India (‘North’, ‘East’, ‘South’, ‘West’, ‘Central’ and ‘Northeast’) (20).

To better understand the diversity of the Indian population and the needs of Indian patients regarding future donor recruitment, we characterized HLA allele and haplotype frequencies of donors registered with DKMS-BMST. We analyzed the HLA data of eight subpopulations, delineated by combining information on geographical origin and native language of both parents of the donor. Four of the eight subpopulations belong to the Indo-Aryan language group (Bengali, Gujarati, Hindi, Marathi) and four to the Dravidian (Kannada, Malayalam, Tamil, Telugu) language family. Furthermore, we analyzed the benefit of DKMS-BMST’s presence and ongoing expansion for Indian patients. Compared to existing studies, our analyses are based on larger and more precisely defined samples.

## 2 Subjects and methods

### 2.1 Samples and HLA typing

**Figure 1:**
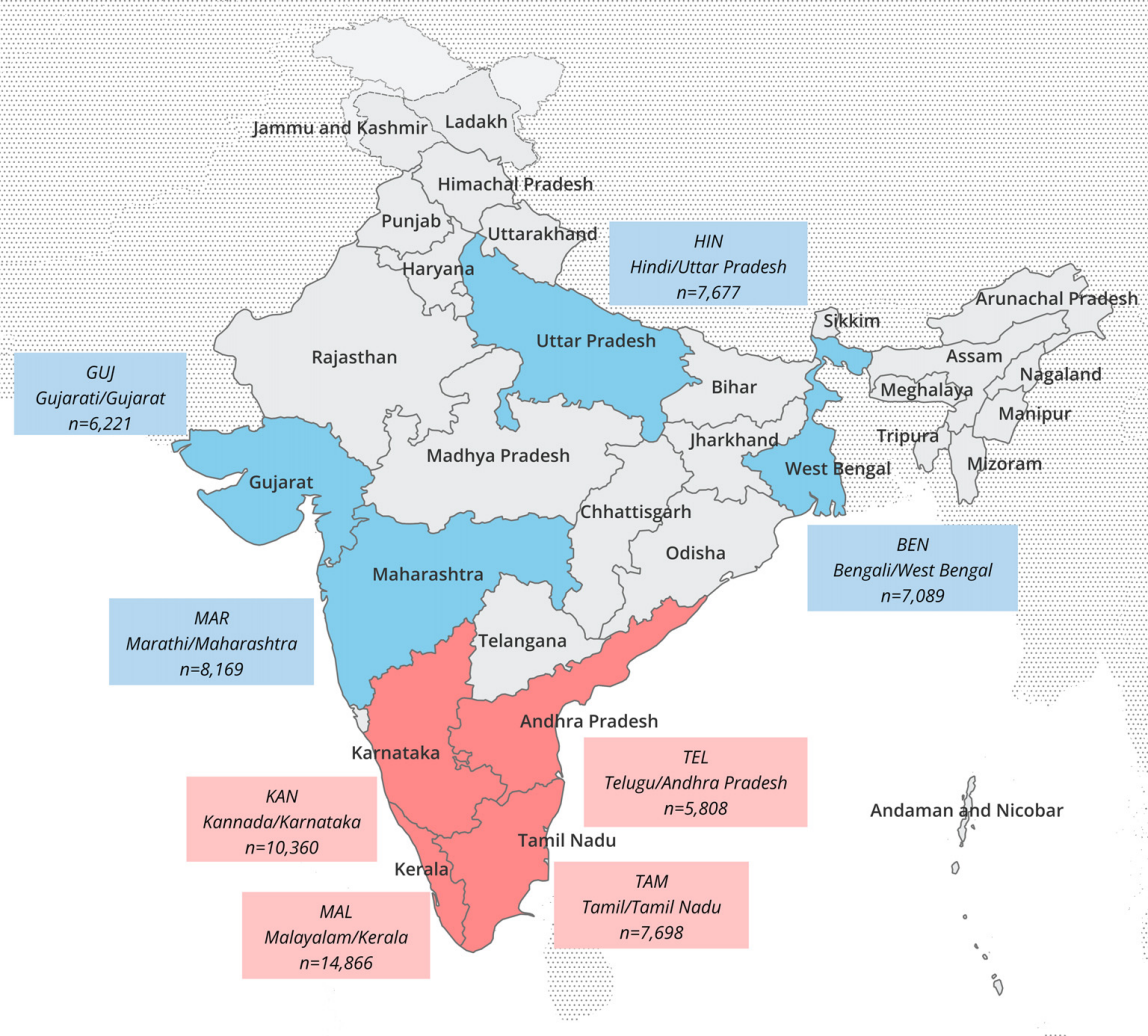
Map of India with subdivisions at state level. The state of origin and native language of both parents of the donors were decisive for the classification of the population samples. Language/state combinations and sizes of the 8 Indian analyzed population samples are indicated in the boxes (red: Dravidian language samples, blue: Indo-Aryan language samples).

As of June 5, 2024, a total of 137,740 active stem cell donors were listed with DKMS-BMST. 6,722 (4.9%) of the donors were excluded from our analyses due to incomplete typing data, 47 (0.03%) due to missing information on state affiliation of the donor and 453 (0.3%) due to the occurrence of new alleles in the typing data (**Supplementary Figure 1**). The current study includes *n*=130,518 registered donors (sample *IND-DKMS)* with HLA-A, -B, -C, -DRB1, -DQB1 and -DPB1 typing data and optional information on the state affiliation of the donors’ parents (**Supplementary Informations 1**, **2**), as well as the native languages of the parents. This information was obtained by donor self-assessment at registration. 69.0% of the donors were male, 31.0% female. With 67.2%, more than two-thirds were between 18 and 30 years old (**Supplementary Figure 2**). The three Indian states with the highest number of donors included in the study were Karnataka (*n*=35,043; 26.8%), Maharashtra (*n*=16,366; 12.5%), and Kerala (*n*=13,723; 10.5%). To achieve a good differentiation of the samples in our analysis, we considered geographical origin and language information of both parents. By setting the lower size limit to *n*=5,000, we obtained 8 population samples, which proceeded into our further analyses: the four Dravidian samples *KAN* (native language: Kannada, state of origin: Karnataka, *n*=10,360), *TAM* (Tamil, Tamil Nadu, *n*=7,698), *TEL* (Telugu, Andhra Pradesh, *n*=5,808), and *MAL* (Malayalam, Kerala, *n*=14,866) and the four Indo-Aryan samples *BEN* (Bengali, West Bengal, *n*=7,089), *GUJ* (Gujarati, Gujarat, *n*=6,221), *HIN* (Hindi, Uttar Pradesh, *n*=7,677), and *MAR* (Marathi, Maharashtra, *n*=8,169) (**Figure 1**, **Supplementary Figure 1**). The complete subsample drawn for the frequency estimation of the 8 population samples thus had a size of *n*=67,888, which corresponds to 52% of sample *IND-DKMS*.

For comparative analyses, we chose two reference populations with stem cell donors of Indian origin registered with DKMS UK (*UK-IND*, *n*=57,218) or DKMS Germany (*DE-IND*, *n*=4,703). Detailed information on the origin of these donors was not available. The donor file of DKMS-BMST (*IND-DKMS*; *n*=130,518) served as a further reference for the matching probability (MP) analyses.

All donor samples were genotyped in a standardized amplicon-based next-generation sequencing workflow on Illumina platforms at DKMS Life Science Lab in Dresden, Germany. Primers were designed to target exons 2 and 3 of HLA-A, -B, -C, -DRB1, -DQB1 and -DPB1 (28, 29). DNA samples were obtained via buccal swabs with the informed consent of the donors. The consent allowed the processing of anonymized donor data for research related to donor search or stem cell donation.

### 2.2 Allele and haplotype frequency estimation

The in-house software Hapl-o-Mat (30, 31) was used to calculate five-locus (HLA-A, -B, -C, -DRB1 and -DQB1) and six-locus (plus HLA-DPB1) haplotype frequencies (HF). Hapl-o-Mat was developed to estimate HF from unphased genotypic data based on an expectation-maximization algorithm (32). To transform donor HLA typing data to a homogeneous output resolution, we used a group representation for synonymous mutations as previously described (33). In brief, alleles differing only in synonymous mutations in the relevant exons (*HLA* class I genes: exon 2 and 3; *HLA* class II genes: exon 2) were joined under a common allele group name and can be distinguished by the trailing letter ‘g’. Haplotype frequencies smaller than 1/(2*n*), the frequency corresponding to a haplotype occurring once in a population sample of size *n*, are of limited information and tend to be artifacts of the estimation process (34, 35). To balance loss of information against the inclusion of artifact haplotypes, haplotypes (sorted most to least frequent) with the lowest frequencies were discarded above the cumulative frequency of 0.995, which means that the HF presented sum up to a cumulative frequency of 99.5%.

Allele frequencies (AF) for all HLA loci were derived from the truncated HF due to typing ambiguities on g-group level in 3,873 of 130,518 individuals (3.0%) and thus also sum up to a cumulative frequency of 99.5%. For computational reasons, 2-locus HF and AF were determined from the full HF set in the linkage disequilibrium calculations.

### 2.3 Linkage disequilibrium, Hardy-Weinberg equilibrium

The linkage disequilibrium (LD) coefficient *D’* was calculated for all 2-locus allele pairs based on the full set of estimated 6-locus HLA haplotype frequencies (36, 37). *P*-values obtained from Fisher’s exact test were subjected to Holm-Bonferroni correction for multiple testing. LD was tested at significance level *α*=0.05. The LD of an allele pair in a specific population was considered to be relevant if it was significant, the associated *D’* value was ≥0.9, and the allele pair had a haplotype frequency of *f*≥0.01.

Tests for significant deviation from Hardy-Weinberg equilibrium (HWE) expectations were carried out with Arlequin v3.5 (38) utilizing an extension of Fisher’s exact test based on Guo and Thompson (39). HWE testing was applied locus-wise using the genotypes on g-group resolution level. Large sample sizes are known to bear the risk to indicate significant results in HWE tests without actual relevance (40). We evaluated deviations from HWE expectations using the effect size statistic *W_n_* (41) and by comparing observed and expected homozygosity of the population samples. *W_n_* values range from 0 to 1. While values near 1 reflect a strong disequilibrium, values below *W_n_* =0.1 were interpreted as an indicator of sufficient agreement with HWE. HWE analyses were not corrected for multiple testing, since this would bias the results toward HWE and lead to a loss of sensitivity.

### 2.4 Genetic distances

Genetic distances (GD) among the eight population and two references samples were assessed as combined Cavalli-Sforza and Edwards chord distances (42). AF were derived from the 6-locus haplotypes taking into account frequencies up to a cumulative frequency of *f*_cum_ *≤* 0.995 and normalized to 1 for the calculations of GD. Locus-wise chord distances were calculated using the formula $d_{j}=\frac{2}{\pi }\;\sqrt{2\cdot (1-\sum_{i=1}^{n}\sqrt{f_{i}\cdot g_{i})}}$ , where *j* is the locus, *n* is the total number of alleles and *f_i_* and *g_i_* are the AF of the two populations at locus j. The global GD for each population pair was calculated as Euclidian overall distance $D=\sqrt{\sum_{j=1}^{m}d_{j}^{2}}$ , where *m* denotes the number of loci considered.

Multidimensional scaling (MDS) was performed in R 3.6.3 (43) using the *cmdscale* function. The goodness-of-fit (GOF) measure, which is based on the eigenvalues of the MDS solution and depends on the number of dimensions used, was applied to evaluate the quality of the distance values’ fit to the graphical representation. GOF values range between 0 and 1; higher values imply a better fit.

To visualize the genetic relationships of the Indian samples to other populations, samples from individuals of Chinese, German and Turkish descent from DKMS Germany, and from individuals of English, Bangladeshi, Pakistani and South East Asian descent from DKMS UK were included in a second GD calculation. These additional reference samples had a size of *n*=4,000 with the exception of the Chinese (*n*=3,705), Southeast Asian (*n*=1,243) and Bangladeshi (*n*=1,829) samples.

### 2.5 Matching probabilities

We defined the 10/10 MP as the likelihood that a random patient from a given population will find at least one fully matched donor from a given donor population. MP were calculated on the basis of 5-locus HF (HLA loci A, B, C, DRB1 and DQB1; 10/10 match) as described before (12, 33, 44, 45). To avoid an influence of different sample sizes on the calculated MP, we drew random samples of *n*=4,000 individuals from the different populations. Only haplotypes with frequencies up to a cumulative frequency of *f*_cum_ *≤* 0.995 were considered and normalized to *f*_cum_=1 prior to calculating the MP (35).

We computed two different scenarios for the 8 Indian population samples and the two references: (I) Patients and donors are from the same population. (II) Patient populations vary, the donor population corresponds to the current composition of the donor file of DKMS-BMST (*IND-DKMS)* and grows with a constant relative population composition. While the first scenario sheds light on population-specific patient benefits through same-population donor recruitment, the second provides information on the extent to which the different populations would benefit from the growth of the registry under the assumption of a constant population ratio.

Based on scenario (I), we additionally calculated the MP for patients in their own donor pool for the 8 Indian populations allowing a single mismatch (HLA loci A, B, C, DRB1 and DQB1; ≥9/10 match).

## 3 Results

### 3.1 Allele and haplotype frequencies

First, we compared the number of distinct alleles that were present at least once in the typing data of the unambiguously resolved genotypes in the 8 Indian population samples (**Supplementary Informations S9**; **S3**-**S8**: not unambiguously present alleles highlighted in gray). The loci with the highest and lowest numbers of alleles were HLA-B (between 76 alleles in *TEL* and 104 in *MAL*) and HLA-DQB1 (between 22 alleles in *BEN* and 29 in *MAL*), respectively.

**Figure 2:**
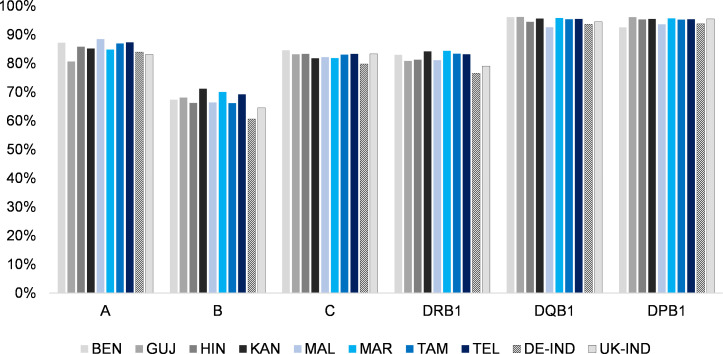
Cumulated frequencies of the respective 10 most frequent alleles of the 8 Indian population samples and the two reference samples for the different HLA loci (HLA-A, -B, -C, DRB1, -DQB1 and -DPB1). Abbreviations for the populations: *BEN*, Bengali/West Bengal; *GUJ*, Gujarati/Gujarat; *HIN*, Hindi/Uttar Pradesh; *KAN*, Kannada/Karnataka; *MAL*, Malayalam/Kerala; *MAR*, Marathi/Maharashtra; *TAM*, Tamil/Tamil Nadu; *TEL*, Telugu/Andhra Pradesh; *DE-IND*, donors of Indian origin registered with DKMS Germany; *UK-IND*, donors of Indian origin registered with DKMS UK.

Accordingly, the cumulative frequencies of the 10 most frequent alleles by population and HLA locus reached the highest values for the HLA-DQB1 locus (between 92.6% in *MAL* and 96.2% in *GUJ*; **Figure 2**, **Table 1**, **Supplementary Information S10**) and the lowest for HLA-B (between 66.1% in *TAM* and 71.1% in *KAN*). No population consistently showed a particularly high or low allelic diversity. The *GUJ* sample, for example, had the lowest cumulated frequencies of the 10 most frequent alleles (“Top 10 alleles”) at HLA loci A and DRB1 (A: 80.7%; DRB1: 80.9%) and the highest cumulated frequencies at the DQB1 and DPB1 loci (DQB1: 96.2%; DPB1: 96.1%).

**Table 1:** Top10 allele frequencies of the 8 donor subsamples of DKMS-BMST.

HLA-A															
BEN		GUJ		HIN		KAN		MAL		MAR		TAM		TEL	
*f*_cum_ (Top 10) =	0.8720	*f*_cum_ (Top 10) =	0.8066	*f*_cum_ (Top 10) =	0.8579	*f*_cum_ (Top 10) =	0.8518	*f*_cum_ (Top 10) =	0.8844	*f*_cum_ (Top 10) =	0.8481	*f*_cum_ (Top 10) =	0.8693	*f*_cum_ (Top 10) =	0.8730
Allele	*f*	Allele	*f*	Allele	*f*	Allele	*f*	Allele	*f*	Allele	*f*	Allele	*f*	Allele	*f*
A*11:01g	0.1902	A*01:01g	0.1697	A*11:01g	0.1445	A*24:02g	0.1381	A*24:02g	0.1903	A*11:01g	0.1527	A*01:01g	0.1610	A*01:01g	0.1706
A*33:03g	0.1889	A*11:01g	0.1517	A*01:01g	0.1375	A*33:03g	0.1373	A*11:01g	0.1449	A*24:02g	0.1320	A*24:02g	0.1562	A*24:02g	0.1445
A*24:02g	0.1255	A*24:02g	0.1013	A*24:02g	0.1368	A*11:01g	0.1334	A*33:03g	0.1362	A*33:03g	0.1317	A*11:01g	0.1380	A*11:01g	0.1356
A*01:01g	0.1093	A*68:01g	0.0934	A*33:03g	0.1175	A*01:01g	0.1282	A*01:01g	0.0808	A*01:01g	0.1190	A*02:11g	0.0813	A*02:11g	0.1133
A*68:01g	0.0581	A*33:03g	0.0664	A*03:01g	0.0757	A*02:11g	0.0926	A*02:01g	0.0751	A*02:11g	0.0996	A*33:03g	0.0782	A*33:03g	0.0766
A*02:03g	0.0569	A*03:01g	0.0625	A*68:01g	0.0726	A*68:01g	0.0595	A*03:01g	0.0707	A*68:01g	0.0564	A*03:01g	0.0679	A*68:01g	0.0675
A*03:01g	0.0509	A*02:01g	0.0471	A*02:11g	0.0656	A*03:01g	0.0583	A*31:01g	0.0520	A*03:01g	0.0492	A*68:01g	0.0578	A*03:01g	0.0542
A*02:11g	0.0417	A*32:01g	0.0432	A*02:01g	0.0424	A*02:01g	0.0404	A*68:01g	0.0516	A*29:01g	0.0437	A*02:01g	0.0503	A*02:01g	0.0479
A*02:01g	0.0259	A*26:01g	0.0393	A*26:01g	0.0365	A*31:01g	0.0323	A*02:11g	0.0472	A*26:01g	0.0337	A*31:01g	0.0416	A*26:01g	0.0322
A*24:07g	0.0248	A*02:11g	0.0320	A*32:01g	0.0288	A*29:01g	0.0316	A*26:01g	0.0356	A*32:01g	0.0301	A*26:01g	0.0369	A*32:01g	0.0306

HLA-B															
BEN		GUJ		HIN		KAN		MAL		MAR		TAM		TEL	
*f*_cum_ (Top 10) =	0.6737	*f*_cum_ (Top 10) =	0.6813	*f*_cum_ (Top 10) =	0.6619	*f*_cum_ (Top 10) =	0.7114	*f*_cum_ (Top 10) =	0.6643	*f*_cum_ (Top 10) =	0.7005	*f*_cum_ (Top 10) =	0.6614	*f*_cum_ (Top 10) =	0.6920
Allele	*f*	Allele	*f*	Allele	*f*	Allele	*f*	Allele	*f*	Allele	*f*	Allele	*f*	Allele	*f*
B*44:03g	0.1282	B*40:06g	0.1250	B*44:03g	0.1071	B*40:06g	0.1244	B*40:06g	0.1061	B*40:06g	0.1418	B*40:06g	0.1087	B*40:06g	0.1227
B*15:02g	0.1098	B*52:01g	0.0907	B*40:06g	0.1034	B*07:05g	0.0887	B*51:01g	0.0810	B*52:01g	0.0906	B*51:01g	0.0910	B*52:01g	0.0957
B*52:01g	0.0823	B*51:01g	0.0897	B*52:01g	0.0916	B*51:01g	0.0801	B*07:02g	0.0707	B*07:05g	0.0821	B*57:01g	0.0819	B*57:01g	0.0867
B*40:06g	0.0810	B*35:03g	0.0707	B*35:03g	0.0873	B*44:03g	0.0753	B*58:01g	0.0687	B*44:03g	0.0796	B*52:01g	0.0759	B*51:01g	0.0862
B*35:03g	0.0655	B*57:01g	0.0669	B*51:01g	0.0626	B*35:03g	0.0717	B*44:03g	0.0652	B*35:01g	0.0682	B*35:03g	0.0676	B*35:03g	0.0823
B*57:01g	0.0530	B*35:01g	0.0629	B*07:02g	0.0475	B*35:01g	0.0677	B*35:03g	0.0607	B*35:03g	0.0649	B*07:05g	0.0532	B*44:03g	0.0610
B*35:01g	0.0523	B*44:03g	0.0545	B*35:01g	0.0473	B*52:01g	0.0636	B*07:05g	0.0559	B*51:01g	0.0613	B*44:03g	0.0492	B*35:01g	0.0491
B*38:02g	0.0356	B*08:01g	0.0470	B*57:01g	0.0434	B*58:01g	0.0554	B*35:01g	0.0534	B*57:01g	0.0412	B*37:01g	0.0484	B*37:01g	0.0419
B*07:02g	0.0339	B*15:02g	0.0404	B*58:01g	0.0388	B*57:01g	0.0472	B*57:01g	0.0518	B*58:01g	0.0398	B*07:02g	0.0435	B*07:05g	0.0342
B*58:01g	0.0320	B*37:01g	0.0334	B*08:01g	0.0330	B*07:02g	0.0374	B*52:01g	0.0508	B*07:02g	0.0310	B*35:01g	0.0419	B*58:01g	0.0322

HLA-C															
BEN		GUJ		HIN		KAN		MAL		MAR		TAM		TEL	
*f*_cum_ (Top 10) =	0.8458	*f*_cum_ (Top 10) =	0.8316	*f*_cum_ (Top 10) =	0.8328	*f*_cum_ (Top 10) =	0.8179	*f*_cum_ (Top 10) =	0.8217	*f*_cum_ (Top 10) =	0.8188	*f*_cum_ (Top 10) =	0.8303	*f*_cum_ (Top 10) =	0.8332
Allele	*f*	Allele	*f*	Allele	*f*	Allele	*f*	Allele	*f*	Allele	*f*	Allele	*f*	Allele	*f*
C*07:01g	0.1489	C*04:01g	0.1393	C*07:01g	0.1419	C*07:02g	0.1397	C*07:02g	0.1392	C*04:01g	0.1308	C*06:02g	0.1554	C*06:02g	0.1584
C*04:01g	0.1173	C*15:02g	0.1361	C*04:01g	0.1196	C*04:01g	0.1378	C*04:01g	0.1187	C*07:02g	0.1177	C*07:02g	0.1391	C*04:01g	0.1236
C*08:01g	0.1123	C*06:02g	0.1291	C*15:02g	0.1169	C*06:02g	0.1002	C*15:02g	0.1081	C*15:02g	0.1172	C*04:01g	0.1085	C*12:02g	0.1085
C*07:02g	0.0937	C*12:02g	0.1071	C*07:02g	0.1110	C*15:02g	0.0999	C*06:02g	0.0928	C*12:02g	0.1094	C*15:02g	0.0976	C*15:02g	0.1056
C*06:02g	0.0879	C*07:02g	0.0913	C*12:02g	0.0983	C*07:01g	0.0909	C*07:01g	0.0922	C*07:01g	0.0984	C*12:02g	0.0849	C*07:02g	0.0912
C*12:02g	0.0866	C*07:01g	0.0874	C*06:02g	0.0959	C*12:02g	0.0753	C*03:02g	0.0692	C*06:02g	0.0906	C*07:01g	0.0743	C*07:01g	0.0834
C*15:02g	0.0763	C*08:01g	0.0434	C*12:03g	0.0554	C*03:02g	0.0572	C*12:02g	0.0599	C*03:02g	0.0413	C*14:02g	0.0566	C*14:02g	0.0506
C*12:03g	0.0447	C*12:03g	0.0404	C*03:02g	0.0389	C*14:02g	0.0446	C*01:02g	0.0594	C*15:05g	0.0395	C*03:02g	0.0399	C*01:02g	0.0482
C*01:02g	0.0440	C*03:02g	0.0288	C*01:02g	0.0304	C*01:02g	0.0395	C*12:03g	0.0486	C*01:02g	0.0377	C*01:02g	0.0377	C*03:02g	0.0322
C*03:02g	0.0339	C*14:02g	0.0287	C*14:02g	0.0244	C*12:03g	0.0328	C*14:02g	0.0337	C*14:02g	0.0362	C*12:03g	0.0362	C*12:03g	0.0315

HLA-DRB1															
BEN		GUJ		HIN		KAN		MAL		MAR		TAM		TEL	
*f*_cum_ (Top 10) =	0.8299	*f*_cum_ (Top 10) =	0.8088	*f*_cum_ (Top 10) =	0.8127	*f*_cum_ (Top 10) =	0.8419	*f*_cum_ (Top 10) =	0.8112	*f*_cum_ (Top 10) =	0.8437	*f*_cum_ (Top 10) =	0.8337	*f*_cum_ (Top 10) =	0.8320
Allele	*f*	Allele	*f*	Allele	*f*	Allele	*f*	Allele	*f*	Allele	*f*	Allele	*f*	Allele	*f*
DRB1*07:01g	0.2344	DRB1*15:02g	0.1356	DRB1*07:01g	0.1790	DRB1*15:01g	0.1576	DRB1*07:01g	0.1570	DRB1*15:01g	0.1707	DRB1*07:01g	0.1637	DRB1*07:01g	0.1709
DRB1*15:02g	0.1797	DRB1*07:01g	0.1269	DRB1*15:02g	0.1082	DRB1*07:01g	0.1366	DRB1*15:01g	0.1308	DRB1*15:02g	0.1588	DRB1*15:01g	0.1206	DRB1*15:02g	0.1292
DRB1*15:01g	0.1059	DRB1*11:01g	0.0883	DRB1*15:01g	0.0987	DRB1*15:02g	0.1253	DRB1*14:04g	0.1060	DRB1*07:01g	0.1309	DRB1*04:03g	0.0980	DRB1*15:01g	0.1002
DRB1*12:02g	0.0829	DRB1*03:01g	0.0833	DRB1*14:04g	0.0805	DRB1*14:04g	0.0804	DRB1*13:02g	0.0751	DRB1*10:01g	0.0886	DRB1*15:02g	0.0878	DRB1*14:04g	0.0805
DRB1*10:01g	0.0539	DRB1*14:04g	0.0804	DRB1*13:01g	0.0713	DRB1*10:01g	0.0752	DRB1*13:01g	0.0745	DRB1*14:04g	0.0866	DRB1*10:01g	0.0869	DRB1*13:01g	0.0725
DRB1*04:03g	0.0482	DRB1*15:01g	0.0743	DRB1*11:01g	0.0701	DRB1*04:03g	0.0650	DRB1*15:02g	0.0630	DRB1*04:03g	0.0515	DRB1*14:04g	0.0780	DRB1*10:01g	0.0686
DRB1*14:04g	0.0465	DRB1*13:01g	0.0728	DRB1*03:01g	0.0675	DRB1*13:01g	0.0611	DRB1*01:01g	0.0561	DRB1*03:01g	0.0451	DRB1*13:01g	0.0680	DRB1*04:03g	0.0677
DRB1*01:01g	0.0265	DRB1*10:01g	0.0560	DRB1*10:01g	0.0561	DRB1*03:01g	0.0561	DRB1*10:01g	0.0559	DRB1*13:01g	0.0447	DRB1*03:01g	0.0641	DRB1*11:01g	0.0498
DRB1*03:01g	0.0261	DRB1*04:03g	0.0535	DRB1*04:03g	0.0461	DRB1*01:01g	0.0521	DRB1*04:03g	0.0558	DRB1*01:01g	0.0364	DRB1*11:01g	0.0403	DRB1*03:01g	0.0465
DRB1*11:01g	0.0259	DRB1*12:02g	0.0378	DRB1*13:02g	0.0352	DRB1*11:01g	0.0325	DRB1*11:01g	0.0369	DRB1*11:01g	0.0304	DRB1*12:02g	0.0263	DRB1*12:02g	0.0460

HLA-DQB1															
BEN		GUJ		HIN		KAN		MAL		MAR		TAM		TEL	
*f*_cum_ (Top 10) =	0.9609	*f*_cum_ (Top 10) =	0.9616	*f*_cum_ (Top 10) =	0.9446	*f*_cum_ (Top 10) =	0.9563	*f*_cum_ (Top 10) =	0.9261	*f*_cum_ (Top 10) =	0.9579	*f*_cum_ (Top 10) =	0.9537	*f*_cum_ (Top 10) =	0.9550
Allele	*f*	Allele	*f*	Allele	*f*	Allele	*f*	Allele	*f*	Allele	*f*	Allele	*f*	Allele	*f*
DQB1*06:01g	0.1916	DQB1*06:01g	0.1784	DQB1*02:01g	0.1987	DQB1*06:01g	0.2427	DQB1*06:01g	0.1449	DQB1*06:01g	0.2874	DQB1*06:01g	0.1771	DQB1*06:01g	0.2059
DQB1*02:01g	0.1901	DQB1*03:01g	0.1592	DQB1*06:01g	0.1725	DQB1*02:01g	0.1465	DQB1*02:01g	0.1215	DQB1*02:01g	0.1369	DQB1*02:01g	0.1434	DQB1*02:01g	0.1312
DQB1*03:01g	0.1277	DQB1*02:01g	0.1549	DQB1*03:01g	0.1284	DQB1*05:01g	0.1289	DQB1*05:03g	0.1208	DQB1*05:01g	0.1280	DQB1*03:02g	0.1239	DQB1*03:01g	0.1247
DQB1*05:01g	0.1222	DQB1*05:03g	0.1047	DQB1*05:03g	0.1087	DQB1*05:03g	0.1065	DQB1*05:01g	0.1160	DQB1*05:03g	0.1166	DQB1*05:01g	0.1120	DQB1*05:03g	0.1056
DQB1*03:03g	0.0821	DQB1*03:02g	0.0982	DQB1*05:01g	0.0786	DQB1*03:02g	0.0904	DQB1*03:01g	0.0984	DQB1*03:02g	0.0752	DQB1*03:01g	0.1073	DQB1*03:02g	0.0996
DQB1*05:03g	0.0745	DQB1*05:01g	0.0868	DQB1*03:02g	0.0749	DQB1*03:01g	0.0754	DQB1*03:02g	0.0877	DQB1*03:01g	0.0723	DQB1*05:03g	0.0946	DQB1*03:03g	0.0910
DQB1*03:02g	0.0736	DQB1*06:03g	0.0757	DQB1*06:03g	0.0744	DQB1*06:03g	0.0623	DQB1*06:03g	0.0771	DQB1*06:03g	0.0455	DQB1*03:03g	0.0880	DQB1*05:01g	0.0843
DQB1*05:02g	0.0582	DQB1*03:03g	0.0617	DQB1*03:03g	0.0545	DQB1*03:03g	0.0522	DQB1*03:03g	0.0709	DQB1*03:03g	0.0440	DQB1*06:03g	0.0687	DQB1*06:03g	0.0731
DQB1*06:03g	0.0257	DQB1*05:02g	0.0262	DQB1*05:02g	0.0326	DQB1*05:02g	0.0298	DQB1*06:09g	0.0513	DQB1*05:02g	0.0383	DQB1*06:02g	0.0217	DQB1*05:02g	0.0232
DQB1*06:02g	0.0153	DQB1*06:02g	0.0157	DQB1*06:04g	0.0211	DQB1*06:09g	0.0216	DQB1*06:02g	0.0374	DQB1*06:02g	0.0138	DQB1*04:02g	0.0170	DQB1*04:02g	0.0163

HLA-DPB1															
BEN		GUJ		HIN		KAN		MAL		MAR		TAM		TEL	
*f*_cum_ (Top 10) =	0.9256	*f*_cum_ (Top 10) =	0.9608	*f*_cum_ (Top 10) =	0.9531	*f*_cum_ (Top 10) =	0.9551	*f*_cum_ (Top 10) =	0.9362	*f*_cum_ (Top 10) =	0.9565	*f*_cum_ (Top 10) =	0.9524	*f*_cum_ (Top 10) =	0.9534
Allele	*f*	Allele	*f*	Allele	*f*	Allele	*f*	Allele	*f*	Allele	*f*	Allele	*f*	Allele	*f*
DPB1*04:01g	0.3139	DPB1*04:01g	0.3908	DPB1*04:01g	0.3969	DPB1*04:01g	0.3431	DPB1*04:01g	0.3212	DPB1*04:01g	0.3410	DPB1*04:01g	0.3249	DPB1*04:01g	0.3235
DPB1*02:01g	0.1789	DPB1*02:01g	0.2181	DPB1*02:01g	0.2074	DPB1*02:01g	0.2328	DPB1*02:01g	0.2391	DPB1*02:01g	0.2338	DPB1*02:01g	0.2664	DPB1*02:01g	0.2684
DPB1*13:01g	0.1269	DPB1*04:02g	0.0806	DPB1*04:02g	0.0765	DPB1*01:01g	0.0624	DPB1*09:01g	0.0685	DPB1*13:01g	0.0671	DPB1*13:01g	0.0631	DPB1*13:01g	0.0641
DPB1*04:02g	0.0829	DPB1*13:01g	0.0611	DPB1*13:01g	0.0670	DPB1*13:01g	0.0581	DPB1*03:01g	0.0542	DPB1*01:01g	0.0570	DPB1*14:01g	0.0590	DPB1*09:01g	0.0577
DPB1*26:01g	0.0584	DPB1*09:01g	0.0548	DPB1*26:01g	0.0533	DPB1*04:02g	0.0547	DPB1*14:01g	0.0533	DPB1*04:02g	0.0525	DPB1*09:01g	0.0553	DPB1*14:01g	0.0519
DPB1*03:01g	0.0522	DPB1*03:01g	0.0473	DPB1*03:01g	0.0433	DPB1*03:01g	0.0542	DPB1*26:01g	0.0491	DPB1*03:01g	0.0513	DPB1*04:02g	0.0447	DPB1*03:01g	0.0508
DPB1*14:01g	0.0444	DPB1*26:01g	0.0462	DPB1*14:01g	0.0416	DPB1*09:01g	0.0475	DPB1*13:01g	0.0484	DPB1*26:01g	0.0493	DPB1*03:01g	0.0435	DPB1*04:02g	0.0469
DPB1*09:01g	0.0333	DPB1*14:01g	0.0349	DPB1*09:01g	0.0390	DPB1*26:01g	0.0464	DPB1*04:02g	0.0451	DPB1*14:01g	0.0465	DPB1*01:01g	0.0425	DPB1*01:01g	0.0388
DPB1*21:01g	0.0182	DPB1*17:01g	0.0173	DPB1*17:01g	0.0154	DPB1*14:01g	0.0438	DPB1*01:01g	0.0448	DPB1*09:01g	0.0444	DPB1*26:01g	0.0403	DPB1*26:01g	0.0358
DPB1*01:01g	0.0165	DPB1*01:01g	0.0098	DPB1*01:01g	0.0126	DPB1*17:01g	0.0120	DPB1*15:01g	0.0125	DPB1*17:01g	0.0137	DPB1*17:01g	0.0127	DPB1*17:01g	0.0155

Allele frequencies are given for gene loci HLA-A, -B, -C, -DRB1, -DQB1, and -DPB1. Coloring was carried out according to the arithmetic means of the allele frequencies of all 8 Indian population samples (*IND-mean*, **Supplementary Tables S3**-**S8**). Darkest blue: alleles with frequency *f* >= 0.1; medium blue: alleles with frequency 0.05<=*f*<0.1; light blue: alleles with frequency 0.035<=*f*<0.05 in the mean distribution. Cumulated frequencies (*f*_cum_) of the Top 10 allele frequencies are indicated. Abbreviations for the populations: *BEN*, Bengali/West Bengal; *GUJ*, Gujarati/Gujarat; *HIN*, Hindi/Uttar Pradesh; *KAN*, Kannada/Karnataka; *MAL*, Malayalam/Kerala; *MAR*, Marathi/Maharashtra; *TAM*, Tamil/Tamil Nadu; *TEL*, Telugu/Andhra Pradesh.

In a comparison of allele frequency ranks between the samples, the results were generally consistent. Notably, for all loci, the five most frequent alleles (“Top 5 alleles”), based on the arithmetic means of the AF of the 8 Indian population samples (from here on referred to as “*IND-mean”*; **Supplementary Information S3**-**S8**) were present within the Top 10 alleles across all ten population samples, with the exception of HLA-B*51:01g (frequency rank #4 in *IND-mean*), which reached only rank #11 in the *BEN* sample. A direct frequency comparison of the union of the respective top 10 allele sets of the 8 Indian subsamples is shown in **Supplementary Information S11**.

For each locus, we determined the 5 allele-population combinations with the largest absolute AF deviations from the *IND-mean* sample (**Table 2**). The largest AF deviation found was that of DQB1*06:01g in the *MAR* sample (Δ*f*=0.087, *f*_MAR_ =0.287, *f*_IND-mean_=0.200). In the 30 allele-population combinations determined, the *BEN* sample appeared by far the most often, namely 11 times, followed by *MAL* with 4 times. Alleles represented more than once were A*33:03g, B*07:05g, C*06:02g, C*07:01g, DRB1*15:02g, DQB1*06:01g, DPB1*02:01 and DPB1*04:01g. A corresponding analysis regarding relative AF deviations from the *IND-mean* sample (larger AF in the numerator, only AF with an absolute deviation of |Δ*f*|≥0.01 considered in order to avoid random findings; **Supplementary Information S12**) revealed the largest relative AF deviation for B*15:32g in the *BEN* sample (*f*_BEN_/*f*_IND-mean_=6.64, *f*_BEN_=0.014, *f*_IND-mean_=0.002). Again, the *BEN* sample appeared most often in the 30 allele-population combinations (12 times), followed by *MAL* (5 times). Alleles A*02:03g, B*07:05g, C*08:01g, DQB1*06:09g and DPB1*01:01g were included in more than one of the 30 allele-population combinations. Four alleles (B*07:05g, B*15:02g, C*08:01g, DRB1*12:02g) were included in the 30 allele-population combinations in both analyses.

**Table 2:** Alleles with the largest absolute frequency differences to the arithmetic means of the 8 Indian population samples (*IND-mean*).

Allele	Sample	*f(*mean)	*f(*sample)	*Δf*	Allele	Sample	*f(*mean)	*f(*sample)	*Δf*
A*33:03g	*BEN*	0.1166	0.1889	0.0723	DRB1*07:01g	*BEN*	0.1624	0.2344	0.0719
A*01:01g	*MAL*	0.1345	0.0808	-0.0537	DRB1*15:02g	*MAL*	0.1234	0.0630	-0.0604
A*33:03g	*GUJ*	0.1166	0.0664	-0.0502	DRB1*15:02g	*BEN*	0.1234	0.1797	0.0562
A*24:02g	*MAL*	0.1406	0.1903	0.0497	DRB1*15:01g	*MAR*	0.1198	0.1707	0.0508
A*02:11g	*TEL*	0.0717	0.1133	0.0417	DRB1*12:02g	*BEN*	0.0327	0.0829	0.0503
B*15:02g	*BEN*	0.0304	0.1098	0.0795	DQB1*06:01g	*MAR*	0.2001	0.2874	0.0873
B*44:03g	*BEN*	0.0775	0.1282	0.0507	DQB1*06:01g	*MAL*	0.2001	0.1449	-0.0552
B*07:05g	*KAN*	0.0432	0.0887	0.0455	DQB1*03:01g	*GUJ*	0.1117	0.1592	0.0475
B*51:01g	*BEN*	0.0727	0.0298	-0.0429	DQB1*02:01g	*HIN*	0.1529	0.1987	0.0458
B*07:05g	*MAR*	0.0432	0.0821	0.0389	DQB1*06:01g	*KAN*	0.2001	0.2427	0.0426
C*08:01g	*BEN*	0.0331	0.1123	0.0793	DPB1*13:01g	*BEN*	0.0695	0.1269	0.0574
C*07:01g	*BEN*	0.1022	0.1489	0.0468	DPB1*04:01g	*HIN*	0.3444	0.3969	0.0525
C*06:02g	*TEL*	0.1138	0.1584	0.0446	DPB1*02:01g	*BEN*	0.2306	0.1789	-0.0517
C*06:02g	*TAM*	0.1138	0.1554	0.0416	DPB1*04:01g	*GUJ*	0.3444	0.3908	0.0463
C*07:01g	*HIN*	0.1022	0.1419	0.0397	DPB1*02:01g	*TEL*	0.2306	0.2684	0.0378

The 5 alleles with the largest absolute values of differences Δ*f*=*f*(sample)-*f*(*IND*-mean) per locus are shown. Abbreviations for the populations: *BEN*, Bengali/West Bengal; *GUJ*, Gujarati/Gujarat; *HIN*, Hindi/Uttar Pradesh; *KAN*, Kannada/Karnataka; *MAL*, Malayalam/Kerala; *MAR*, Marathi/Maharashtra; *TAM*, Tamil/Tamil Nadu; *TEL*, Telugu/Andhra Pradesh.

**Figure 3:**
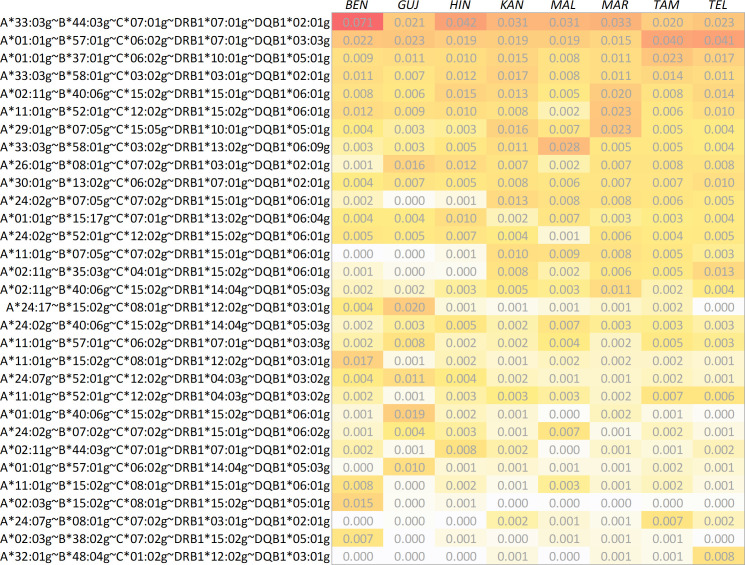
Direct comparison of haplotype frequencies of the 8 Indian population samples. The haplotypes shown correspond to the set union of the respective 10 most frequent haplotypes found in the samples. Haplotypes are ordered by descending arithmetic mean of the eight frequencies. Abbreviations for the populations: *BEN*, Bengali/West Bengal; *GUJ*, Gujarati/Gujarat; *HIN*, Hindi/Uttar Pradesh; *KAN*, Kannada/Karnataka; *MAL*, Malayalam/Kerala; *MAR*, Marathi/Maharashtra; *TAM*, Tamil/Tamil Nadu; *TEL*, Telugu/Andhra Pradesh.

We calculated 5-locus (HLA-A, -B, -C, -DRB1 and -DQB1) and 6-locus (HLA-A, -B, -C, -DRB1, -DQB1 and -DPB1) haplotype frequencies for the 8 Indian population samples and the two reference samples (**Table 3**; a direct frequency comparison of the union of the respective Top 10 HF sets of the 8 Indian subsamples is shown in **Figure 3**; complete 5- and 6-locus HF are given in **Supplementary Informations S13** and **S14**).

**Table 3:** Top 20 5-locus haplotypes of the 8 donor subsamples of DKMS-BMST.

Bengali (West Bengal)	*f*	*f* cum	Gujarati (Gujarat)	*f*	*f* cum
A*33:03g~B*44:03g~C*07:01g~DRB1*07:01g~DQB1*02:01g	0.0707	0.0707	A*01:01g~B*57:01g~C*06:02g~DRB1*07:01g~DQB1*03:03g	0.0229	0.0229
A*01:01g~B*57:01g~C*06:02g~DRB1*07:01g~DQB1*03:03g	0.0224	0.0931	A*33:03g~B*44:03g~C*07:01g~DRB1*07:01g~DQB1*02:01g	0.0205	0.0435
A*11:01g~B*15:02g~C*08:01g~DRB1*12:02g~DQB1*03:01g	0.0169	0.1100	A*24:17~B*15:02g~C*08:01g~DRB1*12:02g~DQB1*03:01g	0.0197	0.0632
A*02:03g~B*15:02g~C*08:01g~DRB1*15:02g~DQB1*05:01g	0.0147	0.1247	A*01:01g~B*40:06g~C*15:02g~DRB1*15:02g~DQB1*06:01g	0.0188	0.0820
A*11:01g~B*52:01g~C*12:02g~DRB1*15:02g~DQB1*06:01g	0.0117	0.1364	A*26:01g~B*08:01g~C*07:02g~DRB1*03:01g~DQB1*02:01g	0.0157	0.0978
A*33:03g~B*58:01g~C*03:02g~DRB1*03:01g~DQB1*02:01g	0.0112	0.1475	A*01:01g~B*37:01g~C*06:02g~DRB1*10:01g~DQB1*05:01g	0.0108	0.1086
A*01:01g~B*37:01g~C*06:02g~DRB1*10:01g~DQB1*05:01g	0.0087	0.1562	A*24:07g~B*52:01g~C*12:02g~DRB1*04:03g~DQB1*03:02g	0.0108	0.1193
A*02:11g~B*40:06g~C*15:02g~DRB1*15:01g~DQB1*06:01g	0.0084	0.1647	A*01:01g~B*57:01g~C*06:02g~DRB1*14:04g~DQB1*05:03g	0.0102	0.1295
A*11:01g~B*15:02g~C*08:01g~DRB1*15:01g~DQB1*06:01g	0.0077	0.1724	A*11:01g~B*52:01g~C*12:02g~DRB1*15:02g~DQB1*06:01g	0.0089	0.1383
A*02:03g~B*38:02g~C*07:02g~DRB1*15:02g~DQB1*05:01g	0.0069	0.1793	A*11:01g~B*57:01g~C*06:02g~DRB1*07:01g~DQB1*03:03g	0.0080	0.1463
A*02:03g~B*15:02g~C*08:01g~DRB1*12:02g~DQB1*03:01g	0.0064	0.1857	A*68:01g~B*40:06g~C*15:02g~DRB1*04:04g~DQB1*03:02g	0.0077	0.1540
A*11:01g~B*44:03g~C*07:01g~DRB1*07:01g~DQB1*02:01g	0.0064	0.1921	A*30:01g~B*13:02g~C*06:02g~DRB1*07:01g~DQB1*02:01g	0.0074	0.1614
A*24:02g~B*15:02g~C*08:01g~DRB1*12:02g~DQB1*03:01g	0.0057	0.1979	A*03:01g~B*35:01g~C*04:01g~DRB1*01:01g~DQB1*05:01g	0.0073	0.1686
A*24:07g~B*35:05g~C*04:01g~DRB1*12:02g~DQB1*03:01g	0.0056	0.2035	A*11:01g~B*40:06g~C*15:02g~DRB1*15:02g~DQB1*06:01g	0.0072	0.1759
A*24:02g~B*52:01g~C*12:02g~DRB1*15:02g~DQB1*06:01g	0.0051	0.2087	A*33:03g~B*58:01g~C*03:02g~DRB1*03:01g~DQB1*02:01g	0.0067	0.1826
A*24:02g~B*44:03g~C*07:01g~DRB1*07:01g~DQB1*02:01g	0.0048	0.2134	A*02:01g~B*52:01g~C*12:02g~DRB1*14:04g~DQB1*05:03g	0.0065	0.1891
A*03:01g~B*35:01g~C*04:01g~DRB1*01:01g~DQB1*05:01g	0.0047	0.2182	A*03:02g~B*18:01g~C*07:01g~DRB1*04:03g~DQB1*03:02g	0.0062	0.1953
A*24:07g~B*52:01g~C*12:02g~DRB1*04:03g~DQB1*03:02g	0.0042	0.2224	A*02:11g~B*40:06g~C*15:02g~DRB1*15:01g~DQB1*06:01g	0.0058	0.2011
A*11:01g~B*35:01g~C*04:01g~DRB1*15:02g~DQB1*06:01g	0.0042	0.2266	A*11:01g~B*44:03g~C*07:01g~DRB1*07:01g~DQB1*02:01g	0.0049	0.2060
A*24:17~B*15:02g~C*08:01g~DRB1*12:02g~DQB1*03:01g	0.0042	0.2308	A*26:63~B*35:03g~C*04:01g~DRB1*11:01g~DQB1*03:01g	0.0048	0.2108

Hindi (Uttar Pradesh)	*f*	*f* cum	Kannada (Karnataka)	*f*	*f* cum
A*33:03g~B*44:03g~C*07:01g~DRB1*07:01g~DQB1*02:01g	0.0419	0.0419	A*33:03g~B*44:03g~C*07:01g~DRB1*07:01g~DQB1*02:01g	0.0308	0.0308
A*01:01g~B*57:01g~C*06:02g~DRB1*07:01g~DQB1*03:03g	0.0191	0.0610	A*01:01g~B*57:01g~C*06:02g~DRB1*07:01g~DQB1*03:03g	0.0187	0.0495
A*02:11g~B*40:06g~C*15:02g~DRB1*15:01g~DQB1*06:01g	0.0154	0.0764	A*33:03g~B*58:01g~C*03:02g~DRB1*03:01g~DQB1*02:01g	0.0169	0.0664
A*33:03g~B*58:01g~C*03:02g~DRB1*03:01g~DQB1*02:01g	0.0122	0.0886	A*29:01g~B*07:05g~C*15:05g~DRB1*10:01g~DQB1*05:01g	0.0161	0.0825
A*26:01g~B*08:01g~C*07:02g~DRB1*03:01g~DQB1*02:01g	0.0116	0.1001	A*01:01g~B*37:01g~C*06:02g~DRB1*10:01g~DQB1*05:01g	0.0152	0.0977
A*01:01g~B*37:01g~C*06:02g~DRB1*10:01g~DQB1*05:01g	0.0105	0.1106	A*02:11g~B*40:06g~C*15:02g~DRB1*15:01g~DQB1*06:01g	0.0130	0.1106
A*01:01g~B*15:17g~C*07:01g~DRB1*13:02g~DQB1*06:04g	0.0104	0.1209	A*24:02g~B*07:05g~C*07:02g~DRB1*15:01g~DQB1*06:01g	0.0126	0.1232
A*11:01g~B*52:01g~C*12:02g~DRB1*15:02g~DQB1*06:01g	0.0101	0.1310	A*33:03g~B*58:01g~C*03:02g~DRB1*13:02g~DQB1*06:09g	0.0106	0.1338
A*02:11g~B*44:03g~C*07:01g~DRB1*07:01g~DQB1*02:01g	0.0081	0.1392	A*11:01g~B*07:05g~C*07:02g~DRB1*15:01g~DQB1*06:01g	0.0097	0.1435
A*24:02g~B*52:01g~C*12:02g~DRB1*15:02g~DQB1*06:01g	0.0070	0.1462	A*11:01g~B*52:01g~C*12:02g~DRB1*15:02g~DQB1*06:01g	0.0084	0.1520
A*11:01g~B*35:03g~C*04:01g~DRB1*11:01g~DQB1*03:01g	0.0064	0.1526	A*02:11g~B*35:03g~C*04:01g~DRB1*15:02g~DQB1*06:01g	0.0080	0.1600
A*24:02g~B*35:03g~C*12:03g~DRB1*13:01g~DQB1*06:03g	0.0054	0.1580	A*30:01g~B*13:02g~C*06:02g~DRB1*07:01g~DQB1*02:01g	0.0078	0.1678
A*24:02g~B*40:06g~C*15:02g~DRB1*14:04g~DQB1*05:03g	0.0053	0.1633	A*26:01g~B*08:01g~C*07:02g~DRB1*03:01g~DQB1*02:01g	0.0073	0.1751
A*11:01g~B*44:03g~C*07:01g~DRB1*07:01g~DQB1*02:01g	0.0052	0.1685	A*32:01g~B*35:01g~C*04:01g~DRB1*11:01g~DQB1*03:01g	0.0062	0.1813
A*30:01g~B*13:02g~C*06:02g~DRB1*07:01g~DQB1*02:01g	0.0052	0.1737	A*24:02g~B*40:06g~C*15:02g~DRB1*15:01g~DQB1*06:01g	0.0050	0.1863
A*33:03g~B*58:01g~C*03:02g~DRB1*13:02g~DQB1*06:09g	0.0051	0.1788	A*31:01g~B*51:01g~C*16:02g~DRB1*01:01g~DQB1*05:01g	0.0047	0.1910
A*24:02g~B*44:03g~C*07:01g~DRB1*07:01g~DQB1*02:01g	0.0045	0.1834	A*02:11g~B*40:06g~C*15:02g~DRB1*14:04g~DQB1*05:03g	0.0047	0.1957
A*03:01g~B*18:01g~C*12:03g~DRB1*11:04g~DQB1*03:01g	0.0041	0.1875	A*11:01g~B*35:01g~C*04:01g~DRB1*01:01g~DQB1*05:01g	0.0041	0.1998
A*24:02g~B*40:06g~C*15:02g~DRB1*15:01g~DQB1*06:01g	0.0038	0.1913	A*03:01g~B*35:01g~C*04:01g~DRB1*01:01g~DQB1*05:01g	0.0038	0.2036
A*01:01g~B*44:03g~C*07:01g~DRB1*07:01g~DQB1*02:01g	0.0037	0.1950	A*02:16g~B*07:05g~C*07:02g~DRB1*15:01g~DQB1*06:01g	0.0037	0.2073

Malayalam (Kerala)	*f*	*f* cum	Marathi (Maharashtra)	*f*	*f* cum
A*33:03g~B*44:03g~C*07:01g~DRB1*07:01g~DQB1*02:01g	0.0309	0.0309	A*33:03g~B*44:03g~C*07:01g~DRB1*07:01g~DQB1*02:01g	0.0328	0.0328
A*33:03g~B*58:01g~C*03:02g~DRB1*13:02g~DQB1*06:09g	0.0279	0.0587	A*29:01g~B*07:05g~C*15:05g~DRB1*10:01g~DQB1*05:01g	0.0233	0.0561
A*01:01g~B*57:01g~C*06:02g~DRB1*07:01g~DQB1*03:03g	0.0190	0.0777	A*11:01g~B*52:01g~C*12:02g~DRB1*15:02g~DQB1*06:01g	0.0225	0.0786
A*11:01g~B*07:05g~C*07:02g~DRB1*15:01g~DQB1*06:01g	0.0087	0.0865	A*02:11g~B*40:06g~C*15:02g~DRB1*15:01g~DQB1*06:01g	0.0197	0.0983
A*33:03g~B*58:01g~C*03:02g~DRB1*03:01g~DQB1*02:01g	0.0084	0.0949	A*01:01g~B*57:01g~C*06:02g~DRB1*07:01g~DQB1*03:03g	0.0153	0.1136
A*24:02g~B*07:05g~C*07:02g~DRB1*15:01g~DQB1*06:01g	0.0082	0.1031	A*02:11g~B*40:06g~C*15:02g~DRB1*14:04g~DQB1*05:03g	0.0113	0.1249
A*01:01g~B*37:01g~C*06:02g~DRB1*10:01g~DQB1*05:01g	0.0081	0.1112	A*01:01g~B*37:01g~C*06:02g~DRB1*10:01g~DQB1*05:01g	0.0110	0.1359
A*01:01g~B*15:17g~C*07:01g~DRB1*13:02g~DQB1*06:04g	0.0071	0.1183	A*33:03g~B*58:01g~C*03:02g~DRB1*03:01g~DQB1*02:01g	0.0110	0.1470
A*24:02g~B*40:06g~C*15:02g~DRB1*14:04g~DQB1*05:03g	0.0071	0.1254	A*11:01g~B*07:05g~C*07:02g~DRB1*15:01g~DQB1*06:01g	0.0084	0.1554
A*24:02g~B*07:02g~C*07:02g~DRB1*15:01g~DQB1*06:02g	0.0067	0.1322	A*24:02g~B*07:05g~C*07:02g~DRB1*15:01g~DQB1*06:01g	0.0081	0.1635
A*29:01g~B*07:05g~C*15:05g~DRB1*10:01g~DQB1*05:01g	0.0066	0.1388	A*26:01g~B*08:01g~C*07:02g~DRB1*03:01g~DQB1*02:01g	0.0072	0.1707
A*31:01g~B*51:01g~C*16:02g~DRB1*01:01g~DQB1*05:01g	0.0065	0.1453	A*30:01g~B*13:02g~C*06:02g~DRB1*07:01g~DQB1*02:01g	0.0068	0.1775
A*30:01g~B*13:02g~C*06:02g~DRB1*07:01g~DQB1*02:01g	0.0061	0.1514	A*24:02g~B*52:01g~C*12:02g~DRB1*15:02g~DQB1*06:01g	0.0064	0.1839
A*03:01g~B*07:02g~C*07:02g~DRB1*15:01g~DQB1*06:02g	0.0057	0.1570	A*02:11g~B*35:03g~C*04:01g~DRB1*15:02g~DQB1*06:01g	0.0061	0.1900
A*32:01g~B*35:01g~C*04:01g~DRB1*11:01g~DQB1*03:01g	0.0054	0.1624	A*32:01g~B*35:01g~C*04:01g~DRB1*11:01g~DQB1*03:01g	0.0053	0.1952
A*02:11g~B*40:06g~C*15:02g~DRB1*15:01g~DQB1*06:01g	0.0050	0.1674	A*33:03g~B*58:01g~C*03:02g~DRB1*13:02g~DQB1*06:09g	0.0052	0.2005
A*24:02g~B*44:03g~C*07:01g~DRB1*07:01g~DQB1*02:01g	0.0050	0.1724	A*24:02g~B*40:06g~C*15:02g~DRB1*15:01g~DQB1*06:01g	0.0048	0.2053
A*24:02g~B*57:01g~C*06:02g~DRB1*07:01g~DQB1*03:03g	0.0048	0.1772	A*02:11g~B*07:05g~C*07:02g~DRB1*15:01g~DQB1*06:01g	0.0043	0.2096
A*11:01g~B*40:06g~C*15:02g~DRB1*14:04g~DQB1*05:03g	0.0048	0.1820	A*11:01g~B*40:06g~C*15:02g~DRB1*15:01g~DQB1*06:01g	0.0041	0.2137
A*11:01g~B*07:05g~C*07:02g~DRB1*14:04g~DQB1*05:03g	0.0044	0.1864	A*11:01g~B*35:01g~C*04:01g~DRB1*01:01g~DQB1*05:01g	0.0040	0.2177

Tamil (Tamil Nadu)	*f*	*f* cum	Telugu (Andhra Pradesh)	*f*	*f* cum
A*01:01g~B*57:01g~C*06:02g~DRB1*07:01g~DQB1*03:03g	0.0399	0.0399	A*01:01g~B*57:01g~C*06:02g~DRB1*07:01g~DQB1*03:03g	0.0415	0.0415
A*01:01g~B*37:01g~C*06:02g~DRB1*10:01g~DQB1*05:01g	0.0227	0.0625	A*33:03g~B*44:03g~C*07:01g~DRB1*07:01g~DQB1*02:01g	0.0227	0.0642
A*33:03g~B*44:03g~C*07:01g~DRB1*07:01g~DQB1*02:01g	0.0195	0.0820	A*01:01g~B*37:01g~C*06:02g~DRB1*10:01g~DQB1*05:01g	0.0169	0.0811
A*33:03g~B*58:01g~C*03:02g~DRB1*03:01g~DQB1*02:01g	0.0141	0.0961	A*02:11g~B*40:06g~C*15:02g~DRB1*15:01g~DQB1*06:01g	0.0140	0.0951
A*02:11g~B*40:06g~C*15:02g~DRB1*15:01g~DQB1*06:01g	0.0079	0.1040	A*02:11g~B*35:03g~C*04:01g~DRB1*15:02g~DQB1*06:01g	0.0129	0.1080
A*26:01g~B*08:01g~C*07:02g~DRB1*03:01g~DQB1*02:01g	0.0076	0.1116	A*33:03g~B*58:01g~C*03:02g~DRB1*03:01g~DQB1*02:01g	0.0115	0.1195
A*24:07g~B*08:01g~C*07:02g~DRB1*03:01g~DQB1*02:01g	0.0072	0.1188	A*11:01g~B*52:01g~C*12:02g~DRB1*15:02g~DQB1*06:01g	0.0100	0.1295
A*30:01g~B*13:02g~C*06:02g~DRB1*07:01g~DQB1*02:01g	0.0067	0.1255	A*30:01g~B*13:02g~C*06:02g~DRB1*07:01g~DQB1*02:01g	0.0098	0.1393
A*11:01g~B*52:01g~C*12:02g~DRB1*04:03g~DQB1*03:02g	0.0066	0.1321	A*32:01g~B*48:04g~C*01:02g~DRB1*12:02g~DQB1*03:01g	0.0082	0.1474
A*24:02g~B*07:05g~C*07:02g~DRB1*15:01g~DQB1*06:01g	0.0065	0.1386	A*26:01g~B*08:01g~C*07:02g~DRB1*03:01g~DQB1*02:01g	0.0076	0.1550
A*11:01g~B*52:01g~C*12:02g~DRB1*15:02g~DQB1*06:01g	0.0057	0.1442	A*02:01g~B*52:01g~C*12:02g~DRB1*15:02g~DQB1*06:01g	0.0071	0.1621
A*11:01g~B*07:05g~C*07:02g~DRB1*15:01g~DQB1*06:01g	0.0054	0.1496	A*24:02g~B*57:01g~C*06:02g~DRB1*07:01g~DQB1*03:03g	0.0066	0.1687
A*33:03g~B*58:01g~C*03:02g~DRB1*13:02g~DQB1*06:09g	0.0052	0.1548	A*11:01g~B*52:01g~C*12:02g~DRB1*04:03g~DQB1*03:02g	0.0064	0.1750
A*24:02g~B*52:01g~C*12:02g~DRB1*04:03g~DQB1*03:02g	0.0052	0.1600	A*01:01g~B*44:03g~C*07:01g~DRB1*07:01g~DQB1*02:01g	0.0053	0.1803
A*32:01g~B*35:01g~C*04:01g~DRB1*11:01g~DQB1*03:01g	0.0051	0.1651	A*24:02g~B*07:05g~C*07:02g~DRB1*15:01g~DQB1*06:01g	0.0049	0.1852
A*03:01g~B*50:01g~C*06:02g~DRB1*07:01g~DQB1*02:01g	0.0050	0.1701	A*24:02g~B*52:01g~C*12:02g~DRB1*15:02g~DQB1*06:01g	0.0047	0.1898
A*29:01g~B*07:05g~C*15:05g~DRB1*10:01g~DQB1*05:01g	0.0047	0.1748	A*68:01g~B*52:01g~C*12:02g~DRB1*11:01g~DQB1*03:01g	0.0045	0.1944
A*11:01g~B*57:01g~C*06:02g~DRB1*07:01g~DQB1*03:03g	0.0046	0.1794	A*02:11g~B*52:01g~C*12:02g~DRB1*15:02g~DQB1*06:01g	0.0043	0.1987
A*02:11g~B*35:03g~C*04:01g~DRB1*15:02g~DQB1*06:01g	0.0046	0.1840	A*01:01g~B*15:17g~C*07:01g~DRB1*13:02g~DQB1*06:04g	0.0043	0.2030
A*24:02g~B*44:03g~C*07:01g~DRB1*07:01g~DQB1*02:01g	0.0045	0.1885	A*31:01g~B*51:01g~C*14:02g~DRB1*13:01g~DQB1*06:03g	0.0042	0.2071

DE-IND	*f*	*f* cum	UK-IND	*f*	*f* cum
A*33:03g~B*44:03g~C*07:01g~DRB1*07:01g~DQB1*02:01g	0.0237	0.0237	A*26:01g~B*08:01g~C*07:02g~DRB1*03:01g~DQB1*02:01g	0.0260	0.0260
A*01:01g~B*57:01g~C*06:02g~DRB1*07:01g~DQB1*03:03g	0.0162	0.0399	A*33:03g~B*44:03g~C*07:01g~DRB1*07:01g~DQB1*02:01g	0.0219	0.0480
A*26:01g~B*08:01g~C*07:02g~DRB1*03:01g~DQB1*02:01g	0.0160	0.0559	A*01:01g~B*57:01g~C*06:02g~DRB1*07:01g~DQB1*03:03g	0.0200	0.0679
A*01:01g~B*37:01g~C*06:02g~DRB1*10:01g~DQB1*05:01g	0.0125	0.0684	A*01:01g~B*37:01g~C*06:02g~DRB1*10:01g~DQB1*05:01g	0.0123	0.0802
A*33:03g~B*58:01g~C*03:02g~DRB1*03:01g~DQB1*02:01g	0.0119	0.0803	A*33:03g~B*58:01g~C*03:02g~DRB1*03:01g~DQB1*02:01g	0.0096	0.0898
A*02:11g~B*40:06g~C*15:02g~DRB1*15:01g~DQB1*06:01g	0.0096	0.0899	A*02:11g~B*40:06g~C*15:02g~DRB1*15:01g~DQB1*06:01g	0.0080	0.0978
A*33:03g~B*58:01g~C*03:02g~DRB1*13:02g~DQB1*06:09g	0.0093	0.0992	A*33:03g~B*58:01g~C*03:02g~DRB1*13:02g~DQB1*06:09g	0.0076	0.1054
A*01:01g~B*08:01g~C*07:01g~DRB1*03:01g~DQB1*02:01g	0.0086	0.1078	A*11:01g~B*52:01g~C*12:02g~DRB1*15:02g~DQB1*06:01g	0.0074	0.1128
A*11:01g~B*52:01g~C*12:02g~DRB1*15:02g~DQB1*06:01g	0.0083	0.1161	A*30:01g~B*13:02g~C*06:02g~DRB1*07:01g~DQB1*02:01g	0.0069	0.1197
A*30:01g~B*13:02g~C*06:02g~DRB1*07:01g~DQB1*02:01g	0.0070	0.1231	A*24:02g~B*35:03g~C*12:03g~DRB1*13:01g~DQB1*06:03g	0.0057	0.1254
A*01:01g~B*15:17g~C*07:01g~DRB1*13:02g~DQB1*06:04g	0.0066	0.1297	A*01:01g~B*57:01g~C*06:02g~DRB1*14:04g~DQB1*05:03g	0.0057	0.1311
A*29:01g~B*07:05g~C*15:05g~DRB1*10:01g~DQB1*05:01g	0.0066	0.1363	A*01:01g~B*15:17g~C*07:01g~DRB1*13:02g~DQB1*06:04g	0.0053	0.1364
A*03:01g~B*07:02g~C*07:02g~DRB1*15:01g~DQB1*06:02g	0.0060	0.1424	A*02:05g~B*50:01g~C*06:02g~DRB1*07:01g~DQB1*02:01g	0.0052	0.1417
A*03:01g~B*35:01g~C*04:01g~DRB1*01:01g~DQB1*05:01g	0.0054	0.1478	A*24:02g~B*08:01g~C*07:02g~DRB1*03:01g~DQB1*02:01g	0.0050	0.1467
A*24:02g~B*40:06g~C*15:02g~DRB1*14:04g~DQB1*05:03g	0.0047	0.1525	A*29:01g~B*07:05g~C*15:05g~DRB1*10:01g~DQB1*05:01g	0.0048	0.1514
A*24:02g~B*07:02g~C*07:02g~DRB1*15:01g~DQB1*06:02g	0.0046	0.1571	A*24:17~B*15:02g~C*08:01g~DRB1*12:02g~DQB1*03:01g	0.0043	0.1557
A*02:05g~B*50:01g~C*06:02g~DRB1*07:01g~DQB1*02:01g	0.0042	0.1613	A*01:01g~B*08:01g~C*07:02g~DRB1*03:01g~DQB1*02:01g	0.0042	0.1600
A*11:01g~B*35:01g~C*04:01g~DRB1*01:01g~DQB1*05:01g	0.0041	0.1654	A*11:01g~B*57:01g~C*06:02g~DRB1*07:01g~DQB1*03:03g	0.0038	0.1637
A*24:02g~B*57:01g~C*06:02g~DRB1*07:01g~DQB1*03:03g	0.0039	0.1693	A*24:02g~B*35:02g~C*04:01g~DRB1*11:04g~DQB1*03:01g	0.0037	0.1674
A*11:01g~B*07:05g~C*07:02g~DRB1*15:01g~DQB1*06:01g	0.0037	0.1730	A*11:01g~B*44:03g~C*07:01g~DRB1*07:01g~DQB1*02:01g	0.0036	0.1710

Indicated are frequencies *f* and cumulated frequencies *f*_cum_. Coloring was carried out according to the frequency distribution of *IND-mean* (see **Supplementary Information S13**). Dark blue: haplotypes with frequency *f*≥0.01; medium blue: haplotypes with frequency 0.005≤*f*<0.01; light blue: haplotypes with frequency 0.0035≤*f*<0.005 in the mean distribution. Abbreviations for the populations: *BEN*, Bengali/West Bengal; *GUJ*, Gujarati/Gujarat; *HIN*, Hindi/Uttar Pradesh; *KAN*, Kannada/Karnataka; *MAL*, Malayalam/Kerala; *MAR*, Marathi/Maharashtra; *TAM*, Tamil/Tamil Nadu; *TEL*, Telugu/Andhra Pradesh; *DE-IND*, donors of Indian origin registered with DKMS Germany; *UK-IND*, donors of Indian origin registered with DKMS UK.

The cumulated frequencies of the 20 most frequent 5-locus haplotypes of the 8 Indian population samples ranged between 18.6% (*MAL*, **Table 3**) and 23.1% (*BEN*). For the two reference samples *DE-IND* and *UK-IND* the corresponding values were 17.3% and 17.1%, respectively.

There were 5 haplotypes with frequencies *f*≥1% and 6 with frequencies between 0.5% and 1% in *IND-mean* (**Supplementary Information S13**). The 5 haplotypes with a frequency ≥1% were found in the Top 20 haplotypes of all 8 samples. The Top 20 haplotypes of the *MAR* and *KAN* samples showed the largest overlap with the Top 20 of *IND-mean* (16 and 15 identical haplotypes, respectively), the *GUJ* and *BEN* samples the lowest (9 identical haplotypes each). Only these two samples included haplotypes in their Top 5 that were not represented in the Top 20 of *IND-mean*, namely A*11:01g~B*15:02g~C*08:01g~DRB1*12:02g~DQB1*03:01g (#3 in BEN, #23 in *IND-mean*) and A*02:03g~B*15:02g~C*08:01g~DRB1*15:02g~DQB1*05:01g (#4 in BEN, #52 in *IND-mean*) for *BEN* and A*01:01g~B*40:06g~C*15:02g~DRB1*15:02g~DQB1*06:01g (#4 in GUJ, #27 in *IND-mean*) for *GUJ*. The Top 20 haplotypes of the two reference samples contained 13 (*DE-IND*) and 12 (*UK-IND*) of the Top 20 haplotypes of *IND-mean*.

In total, 27,366 different 5-locus haplotypes were found in the 8 Indian population samples, only 252 thereof shared by all samples. The lowest cumulated haplotype frequencies of these shared haplotypes were found in the *MAL* (36.4%) and the *BEN* (40.6%) samples, the highest with 43.5% each in *MAR* and *TEL* (**Supplementary Information S15**).

We determined the 5 haplotype-population combinations with the largest absolute HF deviations from the *IND-mean* sample (**Table 4**). The largest HF deviation was seen for A*33:03g~B*44:03g~C*07:01g~DRB1*07:01g~DQB1*02:01g in the *BEN* sample (Δ*f*=0.037, *f*_BEN_=0.071, *f*_IND-mean_=0.034), the by far most frequent haplotype in any of the samples. In the corresponding analysis with relative HF deviations from the *IND-mean* sample (larger HF in the numerator, only HF with an absolute deviation of |Δ*f*|≥0.0025 considered), the haplotype A*11:01g~B*07:05g~C*07:02g~DRB1*15:01g~DQB1*06:01g (rank #14 in *IND-mean*, *f*_IND-mean_=0.005) occupied the first and the third rank as it only reached very small frequencies in the *BEN* and *GUJ* samples (**Supplementary Information S16**).

**Table 4:** Haplotypes with the largest absolute frequency differences to the arithmetic means of the 8 donor subsamples (*IND-mean*).

Haplotype	Sample	*f(*mean)	*f(*sample)	*Δf*
A*33:03g~B*44:03g~C*07:01g~DRB1*07:01g~DQB1*02:01g	BEN	0.0337	0.0707	0.0369
A*01:01g~B*57:01g~C*06:02g~DRB1*07:01g~DQB1*03:03g	TEL	0.0248	0.0415	0.0166
A*33:03g~B*58:01g~C*03:02g~DRB1*13:02g~DQB1*06:09g	MAL	0.0079	0.0279	0.0199
A*24:17~B*15:02g~C*08:01g~DRB1*12:02g~DQB1*03:01g	GUJ	0.0037	0.0197	0.0160
A*01:01g~B*40:06g~C*15:02g~DRB1*15:02g~DQB1*06:01g	GUJ	0.0032	0.0188	0.0156

The 5 haplotypes with the largest absolute values of differences Δ*f*=*f*(sample)-*f*(*IND*-mean) are shown. Abbreviations for the populations: *BEN*, Bengali/West Bengal; *GUJ*, Gujarati/Gujarat; *MAL*, Malayalam/Kerala; *TAM*, Tamil/Tamil Nadu.

Due to their relevance to the field of unrelated HSCT, detailed analyses and characterizations were limited to the 5-locus HF. 6-locus HF included the additional locus HLA-DPB1. Since the cumulative frequencies of the 10 most frequent alleles by population reached the second highest values of all loci (between 92.6% in *BEN* and 96.1% in *GUJ;* **Figure 2**, **Table 1**, **Supplementary Information S10**), the addition of HLA-DPB1 led to a moderate increase in haplotypes (**Supplementary Information S14**). The highest increase in haplotypes by the addition of locus HLA-DPB1 was found for the *MAL* sample (*n_5loc_* = 5,247, *n_6loc_* =9,228, +75.9%), the lowest for the *HIN* sample (*n_5loc_* =4,477, *n_6loc_* =6,752, +50.8%). The frequencies of the first-rank haplotype per population sample ranged from *f*_MAL_=0.013 to *f*_BEN_=0.035, and the cumulated frequencies of the 20 most frequent haplotypes per population sample still ranged from 11.0% (*MAL*) to 15.0% (*BEN*).

### 3.2 Linkage disequilibrium

In total, we found 42 allele pairs that showed a relevant LD (defined as a statistically significant LD (*p*<0.05) with *D*’≥0.9 and *f*(ab)≥0.01) in at least one of the 8 Indian population samples (**Table 5**; complete lists of allele pairs in significant LD and a haplotype frequency that corresponds to at least a fourfold representation in the sample (*f*≥4/2n, where *n* is the sample size) for the 8 population samples are given in **Supplementary Information S17**). Of these 42 pairs, 21 each belonged to the partial haplotype B~C and to the partial haplotype DRB1~DQB1. The number of allele pairs with relevant LD by sample ranged from 22 (*KAN*; 11 B~C, 11 DRB1~DQB1) to 27 (*GUJ*; 13 B~C, 14 DRB1~DQB1). Four of the B~C and 8 of the DRB1~DQB1 allele pairs showed a relevant LD in all 8 samples (**Table 5**).

**Table 5:** Two-locus linkage disequilibria (*LD*) of the 8 Indian donor samples.

Allele pair	*BEN*	*GUJ*	*HIN*	*KAN*	*MAL*	*MAR*	*TAM*	*TEL*
B*07:02g~C*07:02g (*)	x	x	x	x	x	x	x	x
B*07:05g~C*15:05g				x	x	x		
B*08:01g~C*07:02g			x	x		x		x
B*13:01g~C*04:03g		x	x	x	x	x	x	x
B*13:02g~C*06:02g		x		x	x	x	x	x
B*15:02g~C*08:01g (*)	x	x	x	x	x	x	x	x
B*15:17g~C*07:01g	x	x	x		x	x		
B*15:18g~C*07:04g					x			
B*15:32g~C*12:03g	x							
B*27:05g~C*02:02g	x	x						
B*35:01g~C*04:01g	x	x	x	x	x	x	x	
B*37:01g~C*06:02g	x	x	x	x		x	x	x
B*38:02g~C*07:02g	x		x			x		
B*44:03g~C*07:01g	x						x	x
B*48:04g~C*01:02g								x
B*50:01g~C*06:02g		x	x				x	
B*51:01g~C*16:02g		x						
B*52:01g~C*12:02g		x		x	x	x	x	x
B*55:01g~C*01:02g							x	
B*57:01g~C*06:02g (*)	x	x	x	x	x	x	x	x
B*58:01g~C*03:02g (*)	x	x	x	x	x	x	x	x
DRB1*01:01g~DQB1*05:01g (*)	x	x	x	x	x	x	x	x
DRB1*03:01g~DQB1*02:01g (*)	x	x	x	x	x	x	x	x
DRB1*04:01g~DQB1*03:02g							x	
DRB1*04:03g~DQB1*03:02g (*)	x	x	x	x	x	x	x	x
DRB1*04:04g~DQB1*03:02g		x		x				x
DRB1*04:05g~DQB1*04:01g	x							
DRB1*07:01g~DQB1*03:03g					x		x	x
DRB1*08:03g~DQB1*03:01g					x		x	
DRB1*10:01g~DQB1*05:01g (*)	x	x	x	x	x	x	x	x
DRB1*11:01g~DQB1*03:01g (*)	x	x	x	x	x	x	x	x
DRB1*11:04g~DQB1*03:01g		x	x					
DRB1*12:02g~DQB1*03:01g (*)	x	x	x	x	x	x	x	x
DRB1*13:01g~DQB1*06:03g (*)	x	x	x	x	x	x	x	x
DRB1*13:02g~DQB1*06:04g			x		x		x	x
DRB1*13:02g~DQB1*06:09g			x	x	x	x	x	x
DRB1*14:01g~DQB1*05:03g		x	x					x
DRB1*14:04g~DQB1*05:03g (*)	x	x	x	x	x	x	x	x
DRB1*15:01g~DQB1*06:02g	x	x	x	x	x	x	x	
DRB1*15:02g~DQB1*06:01g		x						
DRB1*15:04g~DQB1*05:02g	x							
DRB1*15:06~DQB1*05:02g	x	x	x		x			

Shown is a list of allele pairs found to exhibit relevant LD (*p*<0.05, *D*’≥0.9 and *f*(ab)≥0.01) in at least one of the populations. The “x” marks the populations for which the LD of the respective allele pair was relevant. Allele pairs with relevant *LD* in all 8 samples are marked with (*). *f*(ab) = frequency of the partial haplotype; *D*’=relative LD; Abbreviations for the populations: *BEN*, Bengali/West Bengal; *GUJ*, Gujarati/Gujarat; *HIN*, Hindi/Uttar Pradesh; *KAN*, Kannada/Karnataka; *MAL*, Malayalam/Kerala; *MAR*, Marathi/Maharashtra; *TAM*, Tamil/Tamil Nadu; *TEL*, Telugu/Andhra Pradesh.

### 3.3 Hardy-Weinberg equilibrium

The number of loci with significant deviations from HWE expectations ranged from 1 (HLA-B in the *MAR* sample) to 6 (*GUJ* and *DE-IND*) (**Supplementary Information S18**). However, small effect size values indicated only moderate deviations from HWE. The highest value observed in all samples was *W_n_* =0.016 for HLA-B in the *DE-IND* sample. We found that deviations from HWE expectations indicated an excess of homozygosity for all affected loci. Deviations from HWE in this direction have been shown not to affect significantly the HF estimation using the EM algorithm (46). Overall, the results of the HWE tests do not represent a limitation for the analyses conducted in this work.

### 3.4 Genetic distances

**Figure 4:**
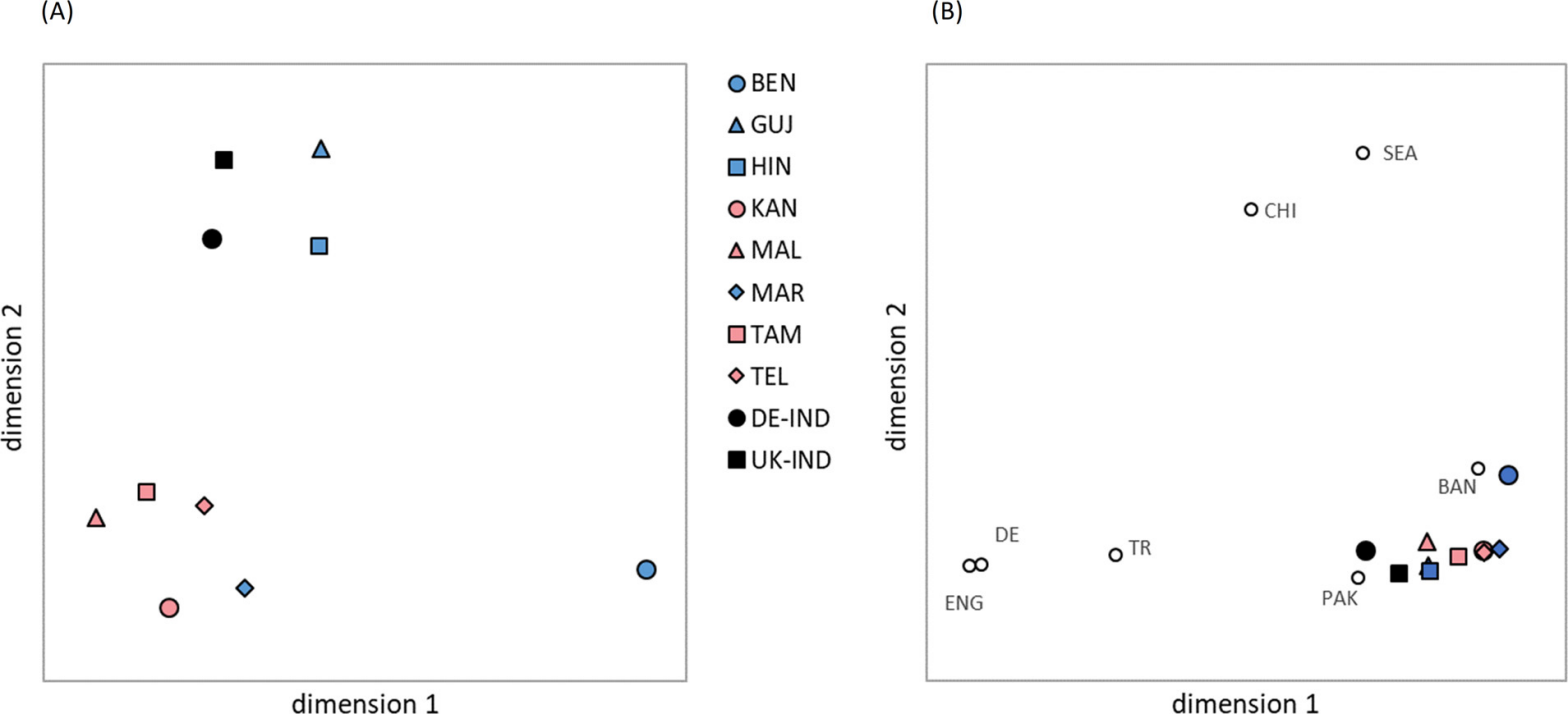
Genetic distances (Euclidian overall distances computed from single-locus Cavalli-Sforza and Edwards chord distances involving loci HLA-A, -B, -C, DRB1, -DQB1 and -DPB1) visualized by multidimensional scaling. **(A)** The 8 Indian population samples and two reference samples *DE-IND* and *UK-IND*. Dimensions 1 and 2 explain 30.9% and 27.8% of the variance, respectively. **(B)** The 8 Indian population samples and two reference samples *DE-IND* and *UK-IND* combined with further reference samples from different ancestries. Dimensions 1 and 2 explain 40.8% and 31.8% of the variance, respectively. Abbreviations for the populations: *BEN*, Bengali/West Bengal; *GUJ*, Gujarati/Gujarat; *HIN*, Hindi/Uttar Pradesh; *KAN*, Kannada/Karnataka; *MAL*, Malayalam/Kerala; *MAR*, Marathi/Maharashtra; *TAM*, Tamil/Tamil Nadu; *TEL*, Telugu/Andhra Pradesh; *DE-IND*, donors of Indian origin registered with DKMS Germany; *UK-IND*, donors of Indian origin registered with DKMS UK; *BAN*, donors of Bangladeshi origin in DKMS UK; *CHI*, donors of Chinese origin in DKMS DE; *DE*, donors of German origin in DKMS DE; *ENG*, donors of English origin in DKMS UK; *PAK*, donors of Pakistani origin in DKMS UK; *SEA*, donors of Southeast Asian origin in DKMS UK; *TR*, donors of Turkish origin in DKMS DE.

The multidimensional scaling of the global GD (**Figure 4A**), which integrated the distance values of all six analyzed HLA loci, indicated a division of the eight Indian population samples into three different clusters. Consistent with observations from previous studies on HLA variation in human populations, the MDS results roughly corresponded to the actual geographic location of the populations (47, 48). A distinct Southern cluster was formed by the four Dravidian population samples *KAN*, *MAL*, *TAM* and *TEL* and the Indo-Aryan *MAR* population sample. The two Indo-Aryan samples *HIN* and *GUJ* grouped together in a Northern cluster, while the *BEN* sample was set distant (Eastern) from all others. The reference samples *DE-IND* and *UK-IND* were located close to the Northern genetic cluster.

Despite the moderate GOF value of 0.587 (variance explained by the two dimensions: 30.9% and 27.8%), the GD visualization effectively represents the pattern of genetic distances calculated among the analyzed samples (**Supplementary Information S19A**). We found the smallest genetic distance between the 8 population samples from India within the Southern cluster of the two-dimensional scaling visualization for *KAN* and *MAR* (*d*=0.178), followed by *TAM*-*TEL* (*d*=0.213) and *KAN*-*TAM* (*d*=0.249). The three greatest distances were all seen for the Bengali sample, namely *BEN*-*MAL* (*d*=0.538), *BEN*-*TAM* (*d*=0.498) and *BEN*-*GUJ* (*d*=0.480). The smallest distance between *BEN* and the remaining samples was *BEN-HIN* (d=0.427). *HIN* and *GUJ*, the two population samples that formed the Northern cluster were also closely related (*d*=0.277), but distant to the remaining population samples from India. Overall, the GD reflected the geographic and linguistic relationships of the 8 populations well, with the notable exception of the Marathi sample which was genetically distinctly closer to the neighboring Dravidian *KAN* population (*d*=0.178) than to the neighboring Indo-Aryan *GUJ* sample (*d*=0.398).

The two reference samples *DE-IND* and *UK-IND* were genetically closer related to each other (*d*=0.193) than to any of the 8 Indian populations (**Supplementary Information S19A**). Among the latter, *UK-IND* exhibited the closest genetic relation to the Northern Indian populations *HIN* (*d*=0.216) and *GUJ* (*d*=0.235). Similarly, *DE-IND* showed the smallest GD to *HIN* (d=0.252), followed by larger distance values to *GUJ* (*d*=0.318) and the South Indian *TAM* (d=0.323) and *MAL* (d=0.326) samples. The largest GD of the two references were determined for *BEN* (*UK-IND*: *d*=0.507, *DE-IND*: *d*=0.492).

The main findings on the genetic overall distances among the 8 population samples and the two references were also reflected in the genetic distances of the individual loci, with minor exceptions in the sequence. For example, for locus HLA-DQB1, the distances for *TEL-GUJ* (*d*=0.084) and *TEL-HIN* (*d*=0.094) were smaller than that for *KAN-TAM* (*d*=0.100) (**Supplementary Information S19A**).

When comparing the Indian population samples with other reference samples of different ancestry, the multidimensional scaling of the global GD (**Figure 4B**; **Supplementary Information S19B**) showed a division into a Southeast/East Asian wing containing the Bangladeshi, Southeast Asian and Chinese samples, and a Northwest Eurasian wing containing the Pakistani, Turkish, English and German samples, again roughly in line with geographic location. The Indian population samples were scaled in a tight cluster between the two wings, with the exception of the *BEN* sample, which revealed a closer relationship with the neighboring Bangladeshi sample than with any of the other Indian samples. From the cluster of Indian population samples, especially the North Indian populations *HIN* and *GUJ* as well as the *DE-IND* and *UK-IND* references were oriented toward the Northwest Eurasian wing and showed a close genetic relationship to the Pakistani sample. The GOF of the graphic representation in **Figure 4B** reached 0.803 (variance explained by the two dimensions: 40.8% and 31.8%).

### 3.5 Matching probabilities

**Figure 5:**
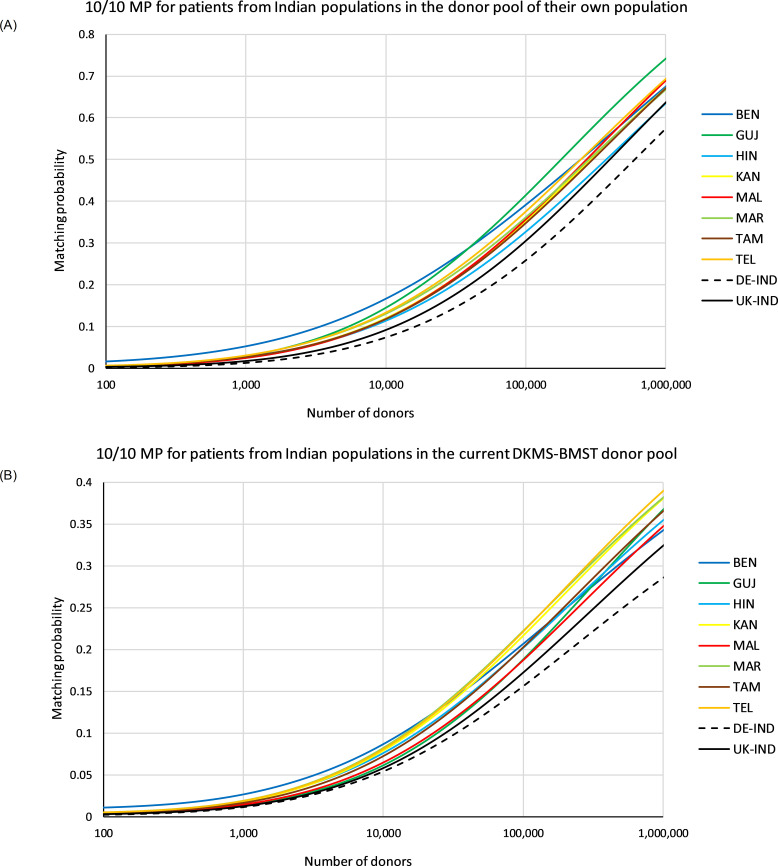
10/10 matching probabilities (MP). **(A)** MP for patients of the different populations in their own donor pool. **(B)** MP for patients of the different populations in a donor population fixed according to the current composition of the DKMS-BMST donor file (sample *IND-DKMS*). Abbreviations for the populations: *BEN*, Bengali/West Bengal; *GUJ*, Gujarati/Gujarat; *HIN*, Hindi/Uttar Pradesh; *KAN*, Kannada/Karnataka; *MAL*, Malayalam/Kerala; *MAR*, Marathi/Maharashtra; *TAM*, Tamil/Tamil Nadu; *TEL*, Telugu/Andhra Pradesh; *DE-IND*, donors of Indian origin in DKMS Germany; *UK-IND*, donors of Indian origin in DKMS UK.

In the first scenario, we calculated 10/10 MP for identical donor and patient populations (**Figure 5A**, see **Supplementary Information S20A** for MP values at different registry sizes). The MP curves of the 5 Indian population samples *KAN*, *MAL*, *MAR*, *TAM* and *TEL* of the Southern cluster in the GD analysis (**Figure 4A**) showed a rather similar course. At a registry size of *n*=100,000, the MP for these 5 populations were between *p*=0.354 (*TAM*) and *p*=0.382 (*TEL*). The MP values of *HIN* remained consistently below these curves (*p*=0.333 at *n*=100,000). The MP curve of the *BEN* sample started with higher values than all other curves, but aligned with the 5 similar curves at registry sizes beyond *n*=100,000 (*p*_BEN_=0.397 at *n*=100,000). For registry sizes of around *n*=2,300 and higher, the MP curve of *GUJ* initially ran between the MP curves of *BEN* and the remaining six Indian subpopulations and above all seven from around *n*=40,000 donors onwards (*p*_GUJ_=0.422 at *n*=100,000). The two reference population samples, *DE-IND* and *UK-IND*, showed lower MP values, as would be expected from samples which represent pools of Indian donors of unspecified origin. While the MP curve of *UK-IND* ran closer below the curve of the *HIN* sample with *p*=0.311 at *n*=100,000, the MP of the *DE-IND* sample only reached *p*=0.265 at that donor file size. In this scenario, MP variation reflects differences between HF distributions of the various samples (**Supplementary Information S22**). In the case of the *BEN* sample, for example, the high MP for very small donor file sizes can be attributed to A*33:03g~B*44:03g~C*07:01g~DRB1*07:01g~DQB1*02:01g. This haplotype block is the by far most frequent among the 10 population samples examined with a frequency of 7.4% (estimated from the reduced sample with *n*=4,000; *f*=7.1% estimated from the original sample with *n*=4,114 donors; **Table 3**).

In the second scenario, 10/10 MP were computed for varying patient populations and a donor pool fixed to a population composition according to the current DKMS-BMST donor file (**Figure 5B**, **Supplementary Information S20B**). Patients from Southern Indian populations had essentially the highest MP at the current DKMS-BMST registry size and above. The Top 3 populations in terms of MP at this scale were *MAR*, *TEL* and *KAN*. At a registry size of *n*=100,000, the MP for these patient populations ranged from p=0.220 (*KAN*) to p=0.226 (*MAR*), and at registry size *n*=1,000,000 from p=0.383 (*KAN*) to p=0.392 (*TEL*). Patients from these Southern Indian populations may benefit from their close genetic relatedness, which increases the chances of finding a matched donor outside their own population. Of this population cluster, only *MAL* showed a lower MP (ranks #8 for n=100,000 and #7 for *n*=1,000,000; **Figure 5B**, **Supplementary Information S20B**). One might assume that population-specific MP reflect the respective donor shares in the *IND-DKMS* sample in this scenario. However, Spearman’s *ρ* revealed a weak negative correlation between donor numbers and MP, e.g. *ρ*=-0.14 for n=100,000. The two reference population samples had the lowest MP values again in this scenario (*p*_UK-IND_=0.176, *p*_DE-IND_=0.159; *n*=100,000).

**Figure 6:**
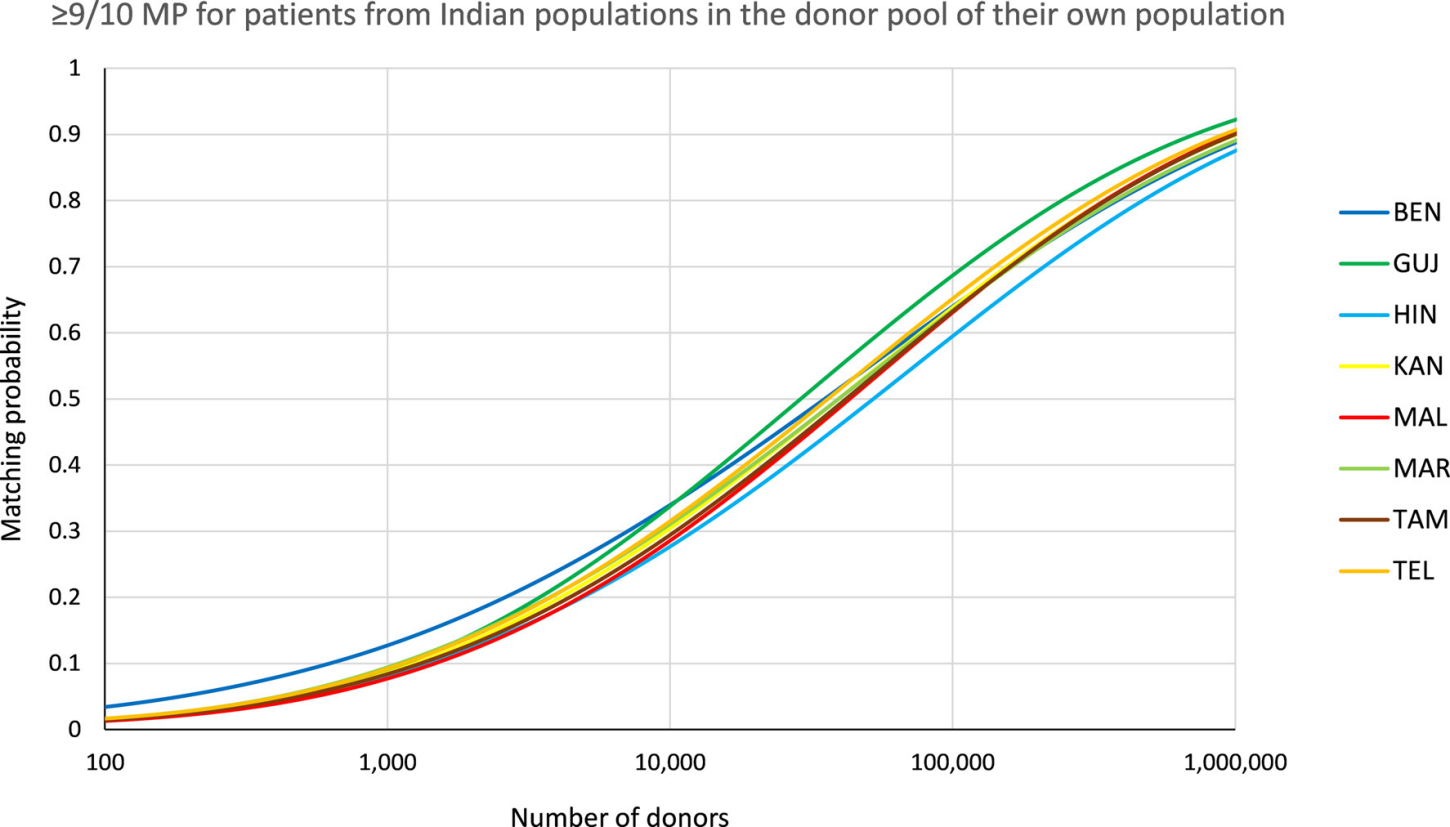
≥9/10 matching probabilities (MP). MP for patients of the different populations in their own donor pool. Abbreviations for the populations: *BEN*, Bengali/West Bengal; *GUJ*, Gujarati/Gujarat; *HIN*, Hindi/Uttar Pradesh; *KAN*, Kannada/Karnataka; *MAL*, Malayalam/Kerala; *MAR*, Marathi/Maharashtra; *TAM*, Tamil/Tamil Nadu; *TEL*, Telugu/Andhra Pradesh.

The permission of one single mismatch (≥9/10 MP) between patient and donor in the scenario with identical patient and donor population increased MP to considerably higher values (**Figure 6**, see **Supplementary Information S21** for MP values at different registry sizes). At a registry size of *n*=100,000, ≥9/10 MP ranged between *p*=0.602 (*HIN*) and p=0.694 (*GUJ*).

## 4 Discussion

In this work, we analyzed HLA data of 8 Indian population samples from a dataset of *n*=130,518 potential hematopoietic stem cell donors registered with DKMS BMST Foundation India (DKMS-BMST), a Bangalore-based donor center with nationwide donor recruitment activities. The populations were delimited according to the state of origin and native language of both parents of the donors. Four of the populations belonged to the Dravidian language family and four to the Indo-Aryan language group. We characterized HLA allele and haplotype frequencies and assessed the benefits of the current and growing registry to Indian patients. The strengths of our study include well-defined, large population samples and comprehensive HLA typing (6 loci at high resolution with an established and quality-proven workflow) (28, 29, 49).

There is only limited published data available on HLA characteristics of Indian subpopulations, especially in high genotyping resolution. The largest study in this regard was conducted on *n*=18,220 individuals from 14 populations by Maiers et al. (21). The analysis included samples in a broad size range between *n*=232 and *n*=5,559, provided by different Indian hematopoietic stem cell donor and cord blood registries and clinical transplant centers. HLA HF served as basis for estimations of patient benefits from regional registry growth. Unlike in our study, the populations were defined by state of origin of the individuals only and not additionally by language. Even if these differences in sample definition should reduce the comparability of our results with those of the study by Maiers et al., there is a remarkable similarity. For the 5 HLA loci A, B, C, DRB1 and DQB1, the Top 10 (Top 5) alleles of the 7 regional populations that were analyzed in both studies overlap by 96.3% (88.6%). Regarding HF, 3 of 7 regional populations share ≥9 of the Top 10 haplotypes. The best correspondence is found in the Karnataka and the Andhra Pradesh samples with all Top 10 haplotypes being identical and in similar order (Spearman’s *ρ*=0.85, each). Dedhia et al. studied AF and HF of HLA loci A, B and DRB1 of individuals speaking Tamil, Telugu, Tulu, Kannada or Malayalam on first-field resolution level (27). Sample sizes ranged from *n*=256 (Tulu) to *n*=463 (Tamil). The comparison of samples for the 4 languages included also in our study (all except Tulu) showed that 98.3% (59/60) of Top 5 allele groups in both studies were consistent. A further study focused on high-resolution HLA-A, -B, -C, -DRB1 and -DQB1 AF and HF in individuals speaking Malayalam, Telugu, Urdu, Kannada or Tamil (26). With exception of the Tamil sample (*n*=7,016), sample sizes were below *n*=400. The AF of this study agree well with our data for the languages analyzed in both studies (all except Urdu). The best agreement is achieved in the Tamil sample, where 24/25 of the Top 5 alleles corresponded in both studies. The differences were slightly larger for the other languages, probably due to small sample sizes. Overall, these evaluations show that our HLA frequency data are in good agreement with previously published data.

A comprehensive review of data in the AFND (20) indicated that of the five HLA haplotypes with a mean frequency of *f≥*1% in the 8 populations from India analyzed in our study, none is unique to Indian populations. A*33:03g~B*44:03g~C*07:01g~DRB1*07:01g~DQB1*02:01g, the most frequent haplotype in the mean distribution and among the Top 3 haplotypes in all 8 populations is also reported from other South Asian and Southeast Asian populations at high frequencies, for example, from South Korea (50), Vietnam (51) and Sri Lanka (52). A closer look at this haplotype block at resolution higher than G groups, however, revealed that in South Asian and especially in Indian population data it usually contains B*44:03:02 and C*07:06, both alleles that are less common in other world populations (20, 23, 24). A geographical distribution across South and Southeast Asian countries is observed for the haplotypes A*01:01g~B*37:01g~C*06:02g~DRB1*10:01g~DQB1*05:01g and A*02:11g~B*40:06g~C*15:02g~DRB1*15:01g~DQB1*06:01g. In addition to South Asia, haplotype A*33:03g~B*58:01g~C*03:02g~DRB1*03:01g~DQB1*02:01g shows a frequent occurrence in East and Southeast Asia, as documented, e.g., for Chinese and Vietnamese populations (51, 53). Haplotype A*01:01g~B*57:01g~C*06:02g~DRB1*07:01g~DQB1*03:03g is not only prevalent in Asian populations, but also globally, with the highest frequency in Tunisia (54). Of the 2-locus haplotypes that showed relevant LD in one or more of the eight Indian population samples (**Table 5**), only three appear to be specific to Indian populations based on a review of the HLA data published in the AFND. These are B*13:01g~C*04:03g, B*48:04g~C*01:02g and DRB1*15:06~DQB1*05:02g, the latter also present in haplotypes of a population from Sri Lanka (52).

The results for the *BEN* sample differed substantially from the 7 other population samples from India. For example, the AF of the *BEN* sample deviated strongly from the mean values of all 8 samples (**Table 2**, **Supplementary Information S12**). Several of the alleles that were more prevalent in the *BEN* sample than in the other samples have their highest frequency in southern Chinese Provinces or countries in Southeast Asia [e.g. B*15:32, A*02:03 and A*33:03 (53, 55–57)], supporting indications of earlier gene flow within these regions (58). Consistently, for each of the 7 other samples, the genetic distance to the *BEN* sample was greater than to all other samples (**Supplementary Information S19A**). In multidimensional scaling, this resulted in an isolated position of the *BEN* sample (**Figure 4A**), not surprising given its geographical location. In comparison with further reference samples from different ancestries, the *BEN* population sample had a closer genetic relationship to the geographically neighboring Bangladeshi sample than to all other Indian samples (**Supplementary Information S19B**) and was more strongly oriented toward the Southeast/East Asian wing of the references in the graphical representation of the GD (**Figure 4B**). With regard to HF, there were also striking deviations of the *BEN* sample from the mean values, whereby *BEN* also stood out here simply because it had by far the most frequent haplotype of all samples (A*33:03g~B*44:03g~C*07:01g~DRB1*07:01g~DQB1*02:01g, *f*_BEN_ =7.1%). The comparatively low haplotypic diversity of the *BEN* sample (**Supplementary Information S22**) is generally advantageous with regard to MP. However, the results in the practice-oriented scenario 2, which simulated the donor search in a growing registry with the current composition of the DKMS-BMST donor database, were rather unfavorable. This also reflects the relatively large genetic distance to the other samples, which means that the Bengali patients benefit less from further donor recruitment according to the current ethnic file composition.

Interestingly, we found the smallest genetic distance of all sample pairs between the Marathi-speaking population from Maharashtra (*MAR*) and the Kannada-speaking population of Karnataka (*KAN*), two geographically neighboring populations belonging to different language families. This indicates that the assumption of language boundaries as marker for genetic differences is not universally valid. In the same way that the extent of admixture or displacement of an indigenous population through historical migration or conquest movements can vary, the language of a conquering or immigrant culture can also be gradually adopted by an existing population without significant changes to the gene pool. Linguistically, Marathi does indeed seem to occupy a special position and is debated as a boundary between the Indo-Aryan and Dravidian languages (59, 60).

The two reference populations *UK-IND* and *DE-IND* were closer to each other in the GD analysis than to the 8 population samples from India. Furthermore, the GD between the reference samples and the individual population samples from India were quite similar, with the exception of *GUJ*, which had a clearly lower GD to *UK-IND* (*d*=0.24) than to *DE-IND* (*d*=0.32; **Supplementary Information S19A**). Furthermore, the reference populations had the lowest MP values of all samples in both scenarios. This is probably due to the fact that these population samples were not as strictly delimited in their ethnic composition as the Indian samples. In addition, there is a clear indication of admixture with the local populations, especially in the haplotype data of *DE-IND*: The very common European haplotype block A*01:01~B*08:01~C*07:01~DRB1*03:01~DQB1*02:01 (53, 61) ranks 8th, while it is found at rank #153 in the *UK-IND* sample and only at rank #8964 in *IND-mean*. Taken together, these results suggest that the ethnic composition of the Indian-origin population in the UK and Germany is similar, with the exception that there are more individuals of Gujarati origin and fewer intermarriages with the local population in the UK. Of note, the haplotype frequencies of a population sample of Indians living in the UK, published in an earlier study, correlated very well with our *UK-IND* data for the 9 most frequent haplotypes (62).

Regarding MP, patients from Southern Indian populations will be the main beneficiaries from further growth of the Bangalore-based DKMS-BMST donor file in its current ethnic composition, presumably because the close genetic relatedness of the populations in this region increases the likelihood of a successful donor search outside the patient’s own population. However, the MP will not diverge too strongly as the respective values range only from *p*=0.345 for *BEN* to *p*=0.392 for *TEL* at donor registry size *n*=1,000,000 (**Supplementary Information S20**). Since DKMS-BMST plans for better nationwide coverage with the opening of regional recruitment offices in different parts of India, we do not see a major risk of undesirable large regional differences in the MP in the future.

MP with different sample sizes are only comparable to a limited extent, since a larger sample size leads to smaller MP values (12). The MP values in the study by Maiers et al. (21) were based on HF of a wide range of sample sizes and represent a variation of our second MP scenario (10/10 MP with equal distribution across the regional groups in a growing Indian registry). Still, they are in good agreement with our results, with a mean MP of *p*=28.1% for a registry size of *n*=100,000. A previous DKMS study had assessed the MP of various populations (10/10 MP, patients and donors from identical populations, corresponding to our first scenario) registered in the UK using samples of *n*=20,000 individuals (63). Accounting for the impact of different sample sizes and different levels of populations structuring, the data on Indian MP presented here are consistent with these earlier findings, which demonstrated how the intra-population diversity impacts the chances of finding a matched donor.

The recent promising evidence concerning the use of PTCy in mismatched unrelated HSCT (6–8) could lead to increased acceptance of mismatches in donor selection in the future, which would particularly benefit populations with high genetic diversity and, more generally, populations that are underrepresented in the global volunteer donor registries. Our analyses showed a considerable increase in MP for the Indian population samples when a single mismatch was permitted (e.g. *HIN* at a registry size of *n*=100,000: *p*_10/10_ = 0.333, *p*_≥9/10_ = 0.602; **Figure 6**, **Supplementary Information S21**).

In the present study, HLA homozygosity exceeding HWE expectations was observed in all populations. These findings may have resulted from non-random mating, as pairs formed from individuals from the same area could be genetically related due to limited migration and decreased influx of new HLA genotypes into each individual population. In addition, unaccounted substructure may also be the cause for reduced heterozygosity in population samples (Wahlund effect).

The high proportion of male donors in the DKMS-BMST donor file deviates remarkably from those found in other DKMS entities (63) and also from the situation in registries worldwide (64). As they are the most likely to actually donate (65), DKMS prioritizes the recruitment of young men. The focus of DKMS India’s offline donor recruitment is on IT companies and technical colleges, which is proving successful in this regard.

Our study is subject to several limitations that may influence the results. First, the definition of distinct populations of adequate sample sizes in a complex, multi-ethnic country like India is always arbitrary to a certain extent. Second, the ancestry assignment of the donors was based on self-assessment during the recruitment process. This routine can cause inaccuracies, particularly for donors of mixed ethnicity. However, our approach of integrating state of origin and native language of the donors’ parents should provide a sufficient accuracy. Third, since donor recruitment takes place particularly in urban areas and mainly reaches younger individuals with an above-average socioeconomic status, stem cell donors do not represent an unbiased sample of the actual population. Fourth, the decision to exclude haplotypes with the lowest 0.5% of cumulative frequencies in order to limit the impact of artifacts of the estimation process implies the acceptance of a certain loss of information. And last, regarding the MP results, it should always be kept in mind that they are based on a simple model that disregards numerous aspects of real-life donor searches, such as donor age, availability or the potential acceptance of donors with selective mismatches.

In summary, we have analyzed HLA allele and haplotype frequencies of stem cell donors registered with DKMS-BMST for 8 Indian subpopulations. The study is the largest of its kind to date. Our results are consistent with published data, but should be more precise due to the larger sample sizes and the exact definition of populations. The frequency distributions obtained are of great relevance for planning the further stem cell donor registry growth in India.

## Data Availability

The aggregated and anonymized data underlying the findings described and used to reach the conclusions of the manuscript are provided in this article and the **Supplementary Material**. Further inquiries can be directed to the corresponding author. Raw data cannot be made publicly available for data protection reasons.
