# Supplementary material for: HLA allele and haplotype frequencies of eight Indian populations based on 130,518 registered stem cell donors
Source: Front Immunol. 2025 Feb 25;16:1528177. doi: 10.3389/fimmu.2025.1528177 (PMC11893841; doi:10.3389/fimmu.2025.1528177)
Supplement: Supplementary file 2 [file DataSheet1.docx]

***Supplemental Figures***


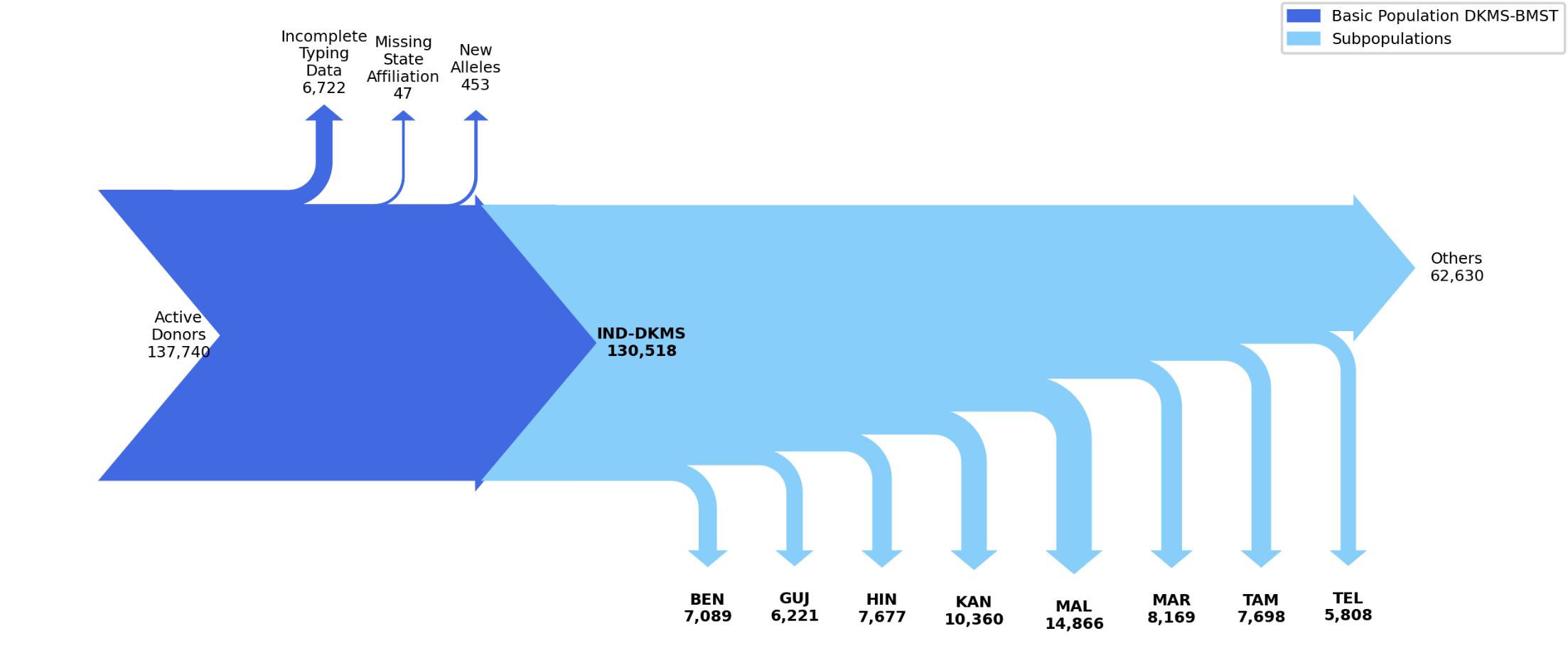


**Supplemental Figure 1.** Overview over the sampling process. Abbreviations for the populations: *BEN*=Bengali/West Bengal, *GUJ*=Gujarati/Gujarat, *HIN*=Hindi/Uttar Pradesh, *KAN*=Kannada/Karnataka, *MAL*=Malayalam/Kerala, *MAR*=Marathi/Maharashtra, *TAM*=Tamil/Tamil Nadu, *TEL*=Telugu/Andhra Pradesh, *IND-DKMS*=DKMS BMST Foundation India donor pool. The numbers of the subjects are given under the respective population names and categories.

**
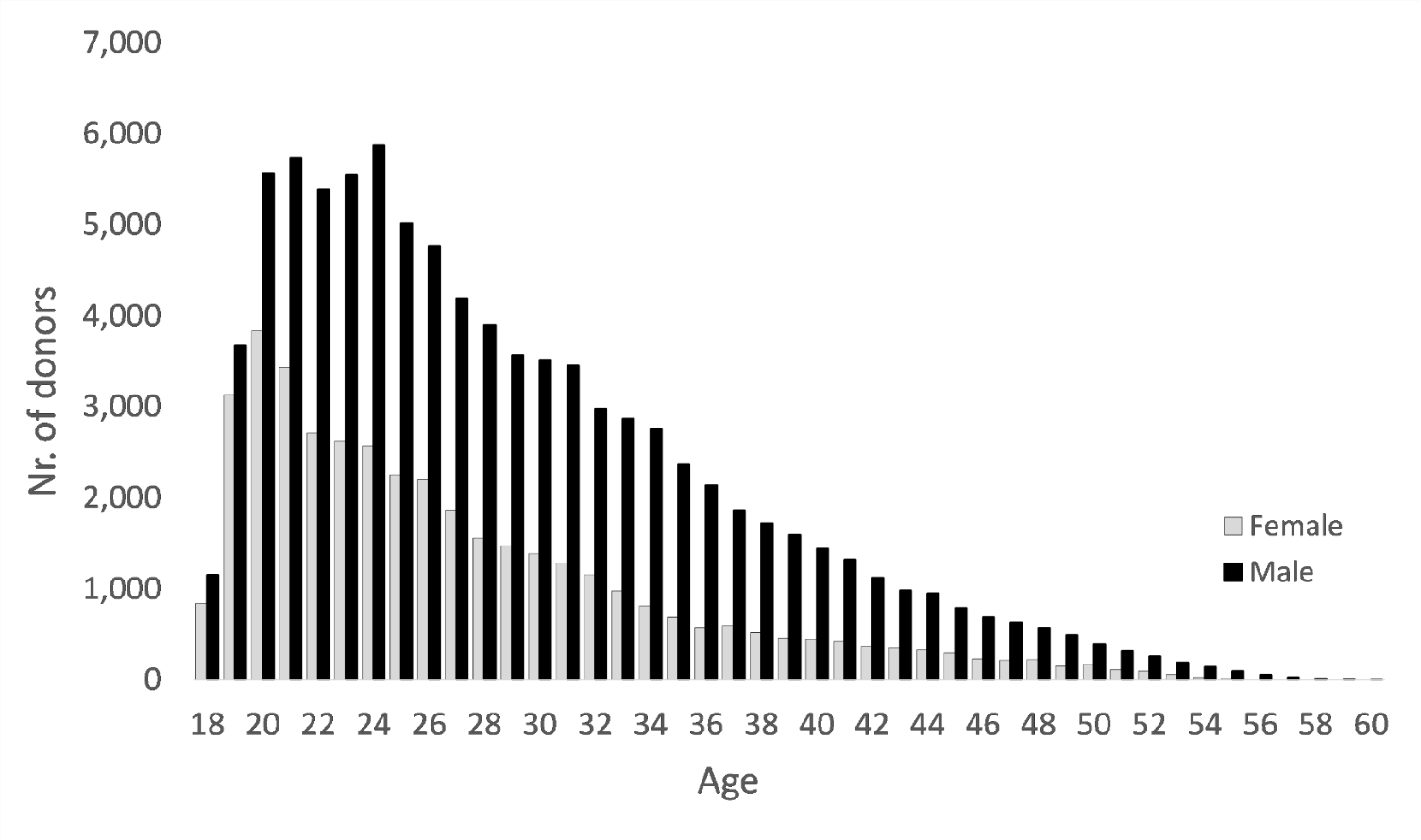
**

**Supplemental Figure 2.** Age-sex distribution in the current DKMS BMST Foundation India donor pool (*IND-DKMS*, *n*=130,518; June 2024).
